# Genomic Survey of *E. coli* From the Bladders of Women With and Without Lower Urinary Tract Symptoms

**DOI:** 10.3389/fmicb.2020.02094

**Published:** 2020-09-04

**Authors:** Andrea Garretto, Taylor Miller-Ensminger, Adriana Ene, Zubia Merchant, Aashaka Shah, Athina Gerodias, Anthony Biancofiori, Stacey Canchola, Stephanie Canchola, Emanuel Castillo, Tasnim Chowdhury, Nikita Gandhi, Sarah Hamilton, Kyla Hatton, Syed Hyder, Koty Krull, Demetrios Lagios, Thinh Lam, Kennedy Mitchell, Christine Mortensen, Amber Murphy, Joseph Richburg, Meghan Rokas, Suzanne Ryclik, Pauline Sulit, Thomas Szwajnos, Manuel Widuch, Jessica Willis, Mary Woloszyn, Bridget Brassil, Genevieve Johnson, Rita Mormando, Laura Maskeri, Mary Batrich, Nicole Stark, Jason W. Shapiro, Cesar Montelongo Hernandez, Swarnali Banerjee, Alan J. Wolfe, Catherine Putonti

**Affiliations:** ^1^Bioinformatics Program, Loyola University Chicago, Chicago, IL, United States; ^2^Department of Biology, Loyola University Chicago, Chicago, IL, United States; ^3^Neuroscience Program, Loyola University Chicago, Chicago, IL, United States; ^4^Niehoff School of Nursing, Stritch School of Medicine, Loyola University Chicago, Maywood, IL, United States; ^5^Department of Microbiology and Immunology, Stritch School of Medicine, Loyola University Chicago, Maywood, IL, United States; ^6^Department of Mathematics and Statistics, Loyola University Chicago, Chicago, IL, United States; ^7^Department of Computer Science, Loyola University Chicago, Chicago, IL, United States

**Keywords:** *E. coli*, UPEC, UTI, urinary microbiome, urobiome

## Abstract

Urinary tract infections (UTIs) are one of the most common human bacterial infections. While UTIs are commonly associated with colonization by *Escherichia coli*, members of this species also have been found within the bladder of individuals with no lower urinary tract symptoms (no LUTS), also known as asymptomatic bacteriuria. Prior studies have found that both uropathogenic *E. coli* (UPEC) strains and *E. coli* isolates that are not associated with UTIs encode for virulence factors. Thus, the reason(s) why *E. coli* sometimes causes UTI-like symptoms remain(s) elusive. In this study, the genomes of 66 *E. coli* isolates from adult female bladders were sequenced. These isolates were collected from four cohorts, including women: (1) without lower urinary tract symptoms, (2) overactive bladder symptoms, (3) urgency urinary incontinence, and (4) a clinical diagnosis of UTI. Comparative genomic analyses were conducted, including core and accessory genome analyses, virulence and motility gene analyses, and antibiotic resistance prediction and testing. We found that the genomic content of these 66 *E. coli* isolates does not correspond with the participant’s symptom status. We thus looked beyond *the E.* coli genomes to the composition of the entire urobiome and found that the presence of *E. coli* alone was not sufficient to distinguish between the urobiomes of individuals with UTI and those with no LUTS. Because *E. coli* presence, abundance, and genomic content appear to be weak predictors of UTI status, we hypothesize that UTI symptoms associated with detection of *E. coli* are more likely the result of urobiome composition.

## Introduction

Urinary tract infections (UTIs) affect about 150 million people per year, mounting large medical costs for individuals and communities (Flores-Mireles et al., 2015). While several different bacterial species are known to cause UTIs, the most frequent culprit is uropathogenic *E. coli* (UPEC), which is thought to account for up to 74.4% of all community-acquired infections (Foxman, 2014). Phylogenomic studies have placed UPEC strains amongst multiple *E. coli* phylotypes (Moriel et al., 2016). Genome sequencing of UPEC isolates has identified numerous virulence factors and pathogenicity islands (Subashchandrabose and Mobley, 2015; Moriel et al., 2016; Terlizzi et al., 2017). Transcriptome analyses have revealed high expression of genes associated with copper efflux and potassium/nickel transport in patients with UTIs (see review; Subashchandrabose et al., 2014). Nevertheless, the UPEC genomic “signature” remains elusive (Johnson et al., 2001, 2015; Luo et al., 2012). At present, there is evidence that the pathogenic potential of *E. coli* cannot be predicted from the presence of specific genetic content, such as a distinct virulence factor. Instead, UTI may be a result of multiple variables (Eto et al., 2007; Ragnarsdóttir et al., 2011; Brubaker and Wolfe, 2017; Schreiber et al., 2017).

Previous evidence indicates that the gut of individuals can be a reservoir for *E. coli* and thus a source of UTI (Yamamoto et al., 1997; Nielsen et al., 2014). Evidence shows that flagellum-mediated motility is key during *E. coli* ascension into and colonization of the urinary tract (Lane et al., 2007). Furthermore, it has been shown that UPEC strains are able to colonize both the gut and urinary tract (Chen et al., 2013). *E. coli* strains isolated from fecal and urine samples of women with UTIs have been found to have similar genomes, and in the absence of symptoms, they cannot be distinguished (Nielsen et al., 2017). This supports the prevailing hypothesis that UTIs can be the result of colonization of the urinary tract by *E. coli* strains from the gut (Yamamoto et al., 1997; Hooton, 2001; Chen et al., 2013; Thänert et al., 2019).

However, the current UTI paradigm does not account for the human urinary microbiota (urobiome) – the newly discovered communities of microbes (bacteria, archaea, protists, fungi, and viruses) that inhabit the bladder. Consequently, traditional clinical care methods remain based on dogma that “urine is sterile.” High throughput DNA sequencing and improved urine culture techniques reveal bacterial DNA and live bacteria, respectively, in catheterized (bladder) urine deemed culture-negative by the standard urine culture method (Fouts et al., 2012; Wolfe et al., 2012; Khasriya et al., 2013; Brubaker et al., 2014; Hilt et al., 2014; Nienhouse et al., 2014; Pearce et al., 2014, 2015; Karstens et al., 2016; Price et al., 2016; Thomas-White et al., 2016, 2017; Coorevits et al., 2017). The urobiome of women without lower urinary tract symptoms (LUTS) often includes species of the genera *Lactobacillus*, *Corynebacterium*, *Gardnerella*, *Staphylococcus*, and *Streptococcus* (see review; Brubaker and Wolfe, 2017). Traditionally, it has been thought that the incidence of *E. coli* in the urinary tract necessitated infection and symptoms, but the discovery of the urobiome and isolation of *E. coli* in asymptomatic people indicates otherwise (Thomas-White et al., 2018b; Price et al., 2020). Moreover, the native microbiota of the bladder has been shown to be distinct from the gastrointestinal tract (Thomas-White et al., 2018a; Price et al., 2020). Thus, a UTI may not always be due to a uropathogen invasion; instead, the urinary tract could itself be the source of UPEC strains. This has been shown previously in individuals with recurrent UTIs (Mysorekar and Hultgren, 2006; Thänert et al., 2019). UTI also could be due to commensal *E. coli* being overly abundant and/or disproportionate to other commensals in the urinary tract.

Here, we present a study of 66 *E. coli* strains isolated from the bladders of women with or without a UTI diagnosis; the latter cohort was further subdivided into women with no lower urinary tract symptoms (no LUTS), overactive bladder (OAB), or urgency urinary incontinence (UUI). Genomes of each of these strains were sequenced. We then compared the evolutionary history, encoded virulence factors, and antibiotic sensitivity of each of these strains in order to identify any factors that distinguish strains associated with patient symptoms. Furthermore, we expanded our analyses to look not only at *E. coli* as the signature of a UTI, but rather the entire microbial community.

## Materials and Methods

### Patient Recruitment

Participant sampling for this study was through several different Institutional Review Board-approved studies [Loyola University Chicago (LU203986, LU205650, LU206449, LU206469, LU207102, and LU207152) and University of California San Diego (170077AW)]. Participants gave verbal and written consent for chart abstraction and urine collection with analysis for research purposes. Members of the Loyola Urinary Education and Research Collaborative (LUEREC), specifically clinicians from the Female Pelvic Medicine and Reconstructive Surgery Center at Loyola University Medical Campus (LUMC), performed recruitment and urine collection. Participants were recruited as a part of separate studies (Hilt et al., 2014; Pearce et al., 2014, 2015; Price et al., 2016, 2020; Thomas-White et al., 2016, 2018a; Dune et al., 2017).

The symptoms of some of the women in the OAB and UUI groups were characterized with the validated Pelvic Floor Distress Inventory symptom questionnaire (Uebersax et al., 1995; Barber et al., 2001). The entire OAB cohort completed the Overactive Bladder Questionnaire (Coyne et al., 2002). For the OAB and UUI cohorts, women with a current UTI (based on urine dipstick test) or a history of recurrent UTI were excluded. For the UTI cohort, participants included women who responded affirmatively to the question “Do you feel you have a UTI?” and completed the UTI symptom assessment (UTISA) questionnaire (Clayson et al., 2005). All UTI samples were from women with cystitis (bladder infection). To be a member of the no LUTS cohort, women had to respond no to this question and be free of LUTS based on the Pelvic Floor Distress Inventory symptom and Overactive Bladder questionnaires. For all studies, exclusion criteria included age of <18 years, pregnancy, catheterization (indwelling or intermittent), or insufficient English skills to complete study measures.

### Urine Collection and EQUC Bacterial Culturing

Urine was collected aseptically via transurethral catheter and was placed in a BD Vacutainer Plus C&S preservative tube for culturing. This technique bypasses the vulva, vagina, and urethra, thus resulting in samples of bacterial isolates specifically from the bladder niche (Wolfe et al., 2012). All samples underwent standard urine culture (SUC), as well as expanded quantitative urine culture (EQUC), as part of the aforementioned studies (Hilt et al., 2014; Pearce et al., 2014, 2015; Price et al., 2016, 2020; Thomas-White et al., 2016, 2018a; Dune et al., 2017). For SUC, 1 μL of urine was inoculated onto 5% sheep blood agar plate (BAP) and MacConkey agar plate (BD BBL prepared plated media), and incubated aerobically at 35°C for 24 h. The detection level was 1000 colony forming unit (CFU) per mL, represented by 1 colony of growth on either plate. EQUC was performed as described previously (Hilt et al., 2014). Briefly, 100 μL of urine was grown under five conditions with BD BBL prepared plated media: (1) BAP in CO_2_ for 48 h, (2) chocolate agar (CHOC) in CO_2_ for 48 h, (3) colistin and nalidixic acid (CNA) agar in CO_2_ for 48 h, (4) CDC anaerobe BAP in an anaerobic jar for 48 h, and (5) BAP in aerobic conditions (BD GasPak Anaerobe Sachets) for 48 h. The detection level was 10 CFU/mL, represented by 1 colony of growth on any of the plates.

Pure cultures were obtained by isolating each morphologically distinct colony type on a different plate of the same medium. Matrix-assisted laser desorption ionization-time of flight (MALDI-TOF) mass spectrophotometry with the MALDI Biotyper 3.0 software program (Bruker Daltonics, Billerica, MA, United States) was used to identify the bacterial strains (see Hilt et al., 2014 for specific protocol, the same as is recommended by the Clinical Laboratory Standards Institute). For some of these isolates, 16S rRNA gene sequencing was performed to verify MALDI-TOF classifications. Previous work by our group showed a concordance between MALDI-TOF and 16S rRNA sequencing classification of urinary isolates (Pearce et al., 2014; Price et al., 2020). Strains identified as *E. coli* via MALDI-TOF were grown from single colonies in lysogeny (LB) broth (10.0 g/L tryptone, 5.0 g/L yeast, 10.0 g/L NaCl). Stocks of each strain were made using 900 μL 50% glycerol mixed with 90 μL *E. coli* cells, and stored at −80°C.

### DNA Extraction and Genome Sequencing

Liquid cultures of each strain (Supplementary Table 1) were grown from freezer stock in LB at 37°C in a shaking (150 rpm) incubator for 12 h. DNA was extracted from liquid culture using the Qiagen DNeasy UltraClean Microbial Kit following the manufacturer’s protocol. DNA concentration was quantified using the Qubit fluorometer. To confirm the MALDI-TOF classification, the 16S rRNA gene sequence was amplified using the 63f and 1387r primers (Marchesi et al., 1998) and sequenced by Genewiz (New Brunswick, NJ, United States), using each primer individually for 2× coverage. The resulting sequences were manually trimmed and paired in Geneious v11.0.5 (Biomatters, Ltd., Auckland, New Zealand) and queried against the NCBI 16S rRNA sequences database via blastn to confirm that they were *E. coli*. DNA libraries were constructed using the Nextera XT DNA Library preparation kit and sequenced on the Illumina MiSeq platform using the MiSeq Reagent Kit v2 (500-cycles) at Loyola University Chicago’s Genomics Facility (Maywood, IL United States). Raw sequencing reads were deposited in NCBI’s SRA database.

### Assembly and Annotation

Raw reads were first trimmed for quality using the tool Sickle v1.33^1^ and then assembled with SPAdes v3.10.1 using the assembly-only option for *k* values of 55, 77, 99, and 127 (Bankevich et al., 2012). Contigs less than 500 nucleotides in length were removed from further consideration. Assembled contigs were then manually inspected and individually queried against the nr/nt database via megablast, confirming that no contaminants were included in the sequence data. The genome coverage of each assembly was calculated using BBMap’s bbwrap and pileup scripts^2^. Each assembly was annotated using the NCBI Prokaryotic Genome Annotation Pipeline (PGAP) v4.7 (Tatusova et al., 2016).

Assemblies were evaluated for additional genomic features. The *E. coli* serotype was determined using SerotypeFinder v2.0 using default parameters (threshold ID = 85%; minimum length = 60%) (Joensen et al., 2015). The EzClermont web application was used to phylotype each genome (Waters et al., 2020). Genome assemblies were scanned for plasmid replicon sequences using PlasmidFinder v2.1 with default settings (threshold ID = 95%; minimum% coverage = 60%) (Carattoli et al., 2014). All genomes are publicly available through NCBI’s Assembly database (Supplementary Table 2). Virulence factors present within each genome were identified using the virulence factor database (VFDB) using VFanalyzer (Liu et al., 2019). Genes for flagellar synthesis, flagellar rotation, chemotactic signal transduction, and chemotactic membrane receptors were identified within the genome sequences using reciprocal blasts and manual curation. Local blast databases were made using the PGAP annotation files and *flg (flgA-flgN), flh (flhA-flhE), fli (fliA, fliC-fliT*, and *fliZ), mot (motA, motB)* and *che (cheA, cheB, cheR, cheW, cheY*, and *cheZ*) genes as well as chemotactic receptors *tap, tar, trg, tsr*, and *aer* were identified. These sequences were manually inspected. Antibiotic resistance prediction was conducted using ResFinder v3.2 using the default settings (threshold ID = 90%; minimum length = 60%) (Zankari et al., 2012).

### Core Genome and Phylogenomic Analysis

Amino acid sequences for each genome’s RefSeq annotation derived by PGAP were concatenated into a single FASTA format file, sorted by length, and clustered using usearch via the cluster_fast method (Edgar, 2010). Sequence identity thresholds of 70, 80, and 90% were tested; the clusters generated for 80% were selected for further analysis. After manual inspection to confirm homologs were properly identified, the average sequence identity was confirmed to be >90%. The final set of clusters were next parsed using Python to generate a gene presence/absence matrix for the genomes. Using this matrix, hierarchical clustering was performed with SciPy^3^ and the Ward variance minimization algorithm. The resulting tree was converted to Newick format and visualized using iTOL (Letunic and Bork, 2016). This matrix also was used to identify genes belonging to the core and accessory genomes for the 66 *E. coli*. For each gene within the core genome set, the sequences were aligned using MAFFT and alignments were manually inspected (Katoh and Standley, 2013). A consensus tree was derived by first concatenating the alignments followed by derivation using FastTree (Price et al., 2010). The resulting tree was visualized using iTOL (Letunic and Bork, 2016).

### Antibiotic Susceptibility Testing

Antibiotics frequently prescribed for UTI treatment were tested (Chu and Lowder, 2018). Using BBL^TM^ Sensi-Discs (Becton, Dickinson and Company; Franklin Lakes, NJ), each strain was tested for its susceptibility to fosfomycin (200 μg, BD 231709), ciprofloxacin (5 μg, BD 231657), amoxicillin with clavulanic acid (20/10 μg; BD 231628), sulfamethoxazole-trimethoprim (23.75/1.25 μg; BD 231536) and cefpodoxime (10 μg; BD 231673). Testing was conducted as follows. Each *E. coli* strain was incubated in 3 mL of LB broth overnight aerobically, shaking at 37°C. 1 mL was spread on a 1.7% LB agar plate using a cell spreader and left to dry for 5 m. Sensi-Discs were then placed on the *E. coli* lawn using sterile forceps and the plates were incubated overnight at 37°C. The clearance diameter was measured for each Sensi-Disc and each antibiotic was tested in triplicate for each *E. coli* strain. Based upon the diameter measured, the manufacturer’s instructions were used to classify strains as resistant, intermediate, or susceptible.

### Comparing Bladder *E. coli* Isolates to Publicly Available Genomes

Each of the 66 bladder *E. coli* genome assemblies was compared to NCBI’s nr/nt database via megablast. The assembled contigs for each genome assembly were concatenated such that the entire genome sequence was queried. The top hit was recorded for each and the source for the top hit was determined from the GenBank record and/or the literature.

### Statistical Analysis of Communities

The CFU/mL count for all bacteria in each of the 63 independent samples containing one of the sequenced *E. coli* genomes were examined. The individual contributing 3 isolates to our collection of *E. coli* genomes (UMB6653, UMB6721, and UMB7431) was removed from consideration as they are not independent samples. In addition to these data, we included CFU/mL counts for the urobiomes that we have previously characterized from samples of women with UTI symptoms and women without LUTS. In total, this resulted in 109 participants with UTI symptoms and 90 participants with no LUTS. CFU/mL data was recorded at the genus level rather than the species level for our analysis. To analyze prevalence of UTI among participants, a multiple logistic regression model (Cramer, 2002) was used. We treated the no LUTS group as our baseline category for the UTI group. For each participant, abundances of 27 different bacteria were measured; genera appearing in only one sample were removed from consideration. Abundance of these 27 species of bacteria were treated as covariates in our model. Akaike Information Criterion (AIC) (Akaike, 1974; Aho et al., 2014) was used to reduce the full model. All analyses were conducted in R^4^.

## Results

Sixty-six *E. coli* isolates were investigated here, each isolated using the EQUC method from catheterized urine samples from women with (*n* = 42) or without (*n* = 24) a clinical diagnosis of UTI. These isolates were collected from 64 different women as one individual with a recurrent UTI was sampled three times [*E. coli* strains UMB6653 (first visit), UMB6721 (second visit), and UMB7431 (third visit)]. The non-UTI group included asymptomatic ‘healthy’ women [the “no LUTS” group (*n* = 6)] as well as women with OAB (*n* = 5) or UUI (*n* = 13). Supplementary Table 1 lists each isolate and participant symptom diagnosis, as well as the abundance of *E. coli* relative to isolates of other species within the catheterized urine specimens. These data revealed that the abundance of *E. coli* does not strictly relate to diagnostic group, as the UTI group included 6 individuals with low abundances (<10,000 CFU/mL) of *E. coli* within their bladder microbiota, whereas the non-UTI group included 9 individuals with high *E. coli* abundances (≥100,000 CFU/mL).

Whole genome sequencing, genome assembly, and annotation were performed for each isolate (see section “Materials and Methods”). Supplementary Table 2 lists the genome assembly statistics. The genomes ranged in size from 4.6 to 5.6 Mbp. Each genome is represented by long, high quality contig sequences. The average assembly consisted of 73 contigs, ranging from *E. coli* isolate UMB6454 (33 contigs) to *E. coli* isolate UMB2019 (161 contigs). The serotype and phylotypes were determined for each strain (Supplementary Table 1). The genomes represent 39 different serotypes. While most (*n* = 36) isolates belong to the B2 phylotype, 20 isolates belong to phylotype D, 5 to phylotype A, 4 to phylotype B1, and 1 to phylotype F. 59 of the 66 genomes included a contig(s) with a replicon sequence, suggesting that many isolates included plasmid sequences (Supplementary Table 2). Contigs showing similarity to the Col156, IncFII, and IncFIB plasmid replicons were the most abundant, with 28, 45, and 41 isolates harboring these plasmids, respectively.

The core genome of these isolates contained 2,814 genes. This is significantly smaller than the pangenome, which contained an additional 10,182 genes present in one or more of the isolates. However, 28% of the pangenome consists of genes that were only found within one of the 66 *E. coli* genomes, reinforcing prior claims that *E. coli* has an open genome (Rasko et al., 2008).

**FIGURE 1:**
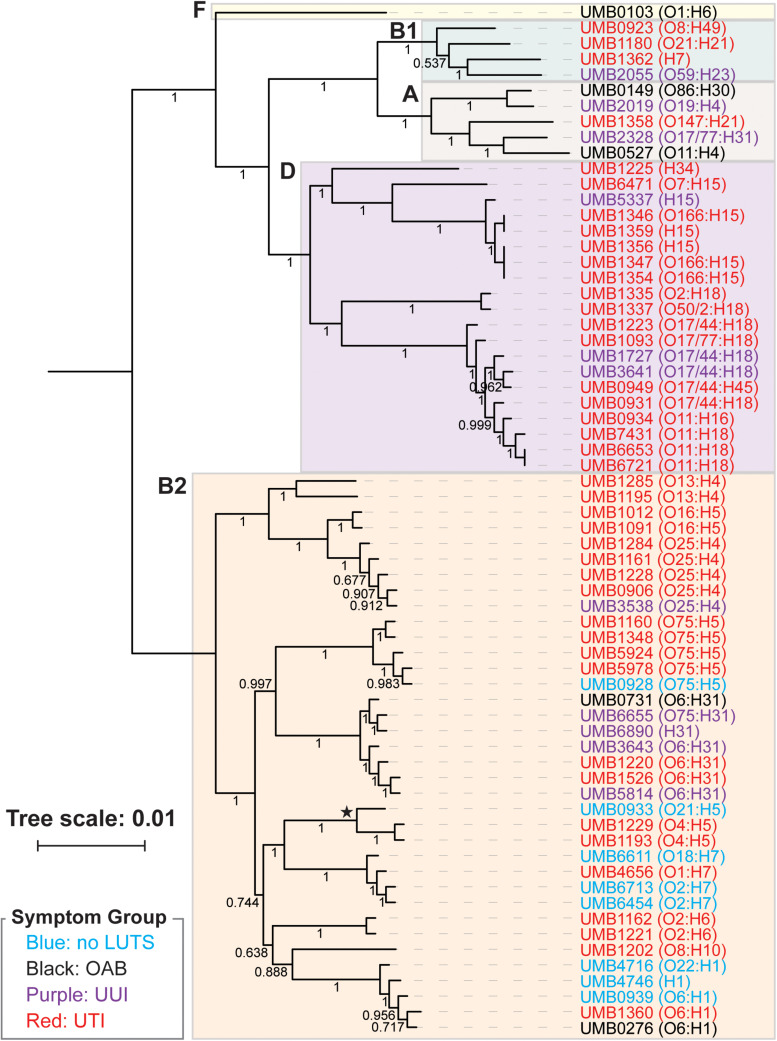
Phlyogenomic relationship of the 66 UTI (red) and non-UTI *E. coli* isolates from the bladder. Non-UTI symptom groups are indicated by color. Phylotypes are indicated by the colored boxes. The branch labeled with a star indicates the 3 isolates of the subphylotype B2_2_. Serotypes for each isolate are listed next to the isolate name. Branch supports are shown.

From the core genome, we constructed a phylogenomic tree (Figure 1). The 66 isolates belong to two distinct clades: the isolates belonging to the B2 phylotype and the isolates belonging to the A, B1, D, and F phylotypes. Symptom status was not associated with these clades; there is no evolutionary distinction between *E. coli* isolates associated with UTI symptoms and those that are not. While the no LUTS isolates were only found within the bottom clade, the sample size is too small to draw conclusions from this observation. In addition to the clear distinction between phylotypes, members of the same serotype often had the most similar core genome sequences.

**FIGURE 2:**
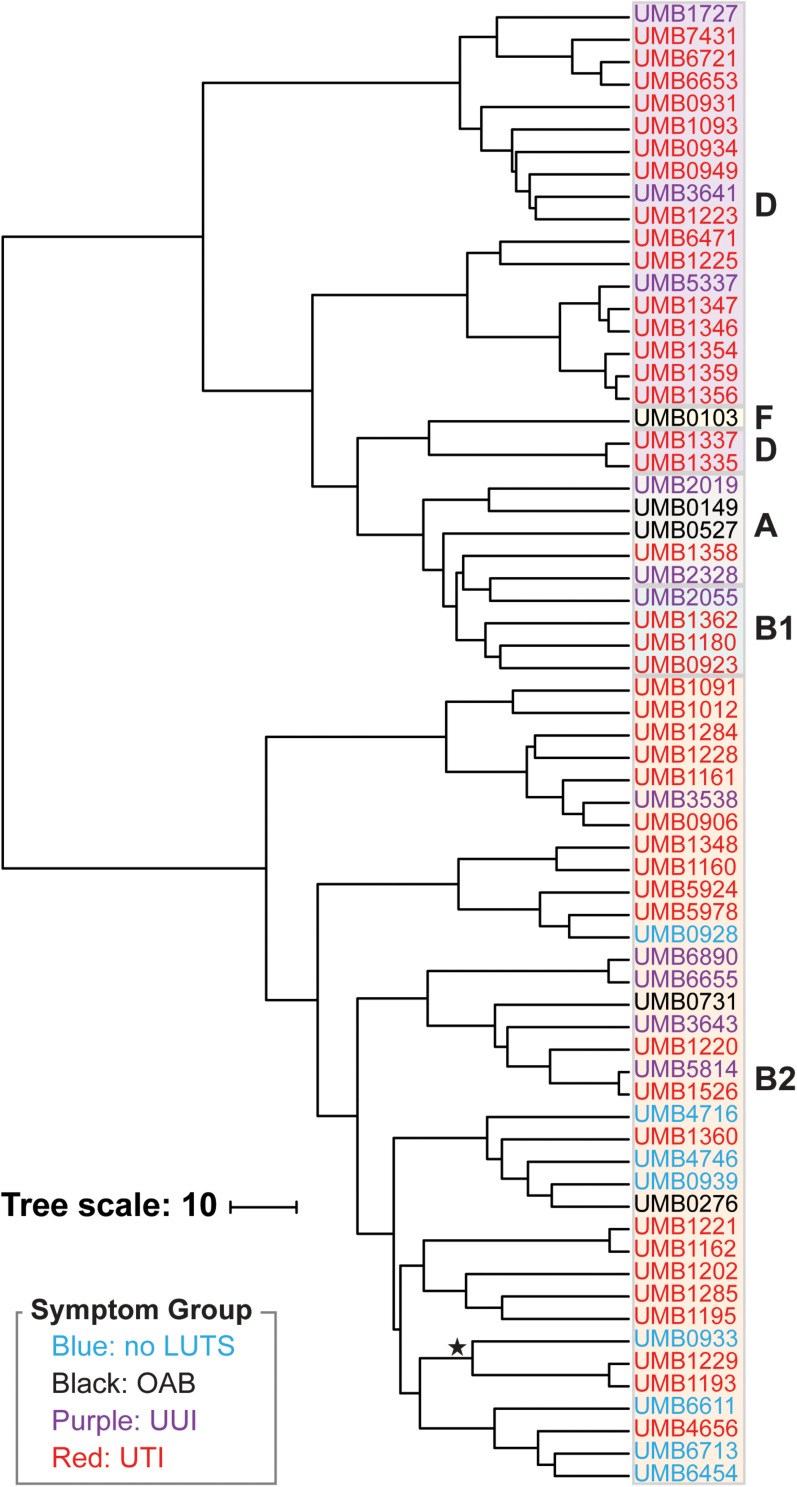
Hierarchical clustering of the 66 E. coli genomes based on gene content. Symptom groups are indicated by color. Phylotypes are indicated by the colored boxes. The branch labeled with a star indicates the 3 isolates of the subphylotype B2_2_.

Next we performed hierarchical clustering of the genes from the pangenome to ascertain if genome content was associated with symptom status (Figure 2). Here, genomes that share more genes in common cluster together. Again, we observed two major clades. Each isolate clustered within the same major clade based on gene content (Figure 2) as it did based on the core genome sequence (Figure 1). The clading structure, however, is not identical; for example, the single isolate in phylotype F, *E. coli* UMB0103, is no longer distinct from the isolates of phylotype D. Further comparison of these two trees reveals that some isolates are more similar with respect to their core gene sequences than they are with respect to the accessory genes that they contain. Using the gene presence matrix, the correlation between each gene’s presence/absence in the 66 *E. coli* genomes and the patient’s clinical diagnosis was calculated. Genes were found to have no or a weak correlation (*r* < 0.5) to clinical diagnosis.

From the accessory genome alone, we first considered the 770 genes present in ≥80% of the UTI genomes and found that these genes were often present (≥50%) in the genomes from participants of the non-UTI symptom groups. There are no genes frequently present (≥80%) in the UTI genomes that are also absent from the non-UTI genomes. Only the gene that encodes the anti-adaptor protein IraD, which inhibits RssB-mediated degradation of RpoS (Battesti et al., 2011; Park et al., 2017), was frequently present in the UTI genomes (86%) and found in only 50% of the non-UTI genomes. All other genes meeting the ≥80% threshold of presence within the UTI genomes were also present in more than half of the non-UTI genomes. In contrast, three genes were underrepresented within the no LUTS group. The genes that encode the type II toxin-antitoxin system YhaV/PrlF and the type I toxin Hok were frequently present in the UTI genomes (≥85%). The genes that encode the YhaV/PrlF system were present in all of the genomes from OAB and UUI participants (*n* = 18) and Hok was present in most of the OAB and UUI participants (*n* = 15).

To further investigate the putative pathogenicity of the isolates, each genome was queried against the virulence factor database (VFDB), identifying 242 different virulence genes (associated with 99 virulence factors) amongst the 66 isolates (Supplementary Table 3). The only virulence factors found in all 42 UTI genomes were also found in all non-UTI (no LUTS, OAB, and UUI) genomes and included: Hemorrhagic *E. coli* pilus (related genes *hcpA* and *hcpC*), Type I fimbriae (related genes *fimF*, *fimG*, and *fimH*), enterohemorrhagic *E. coli* autotransporter B (related gene *ehaB*), and invasion of brain endothelial cells (related genes *ibeB* and *ibeC*). While there were no virulence factors found frequently and exclusively in the UTI isolates, the *E. coli* laminin-binding fimbriae (ELF) and non-LEE type III secretion system effector (TTSS) virulence factors were often present (30 and 40%, respectively) in UTI isolates and absent in all of the no LUTS isolates. However, genes related with these virulence factors were identified in OAB and UUI genomes. No association between virulence factors and symptom state be it UTI/non-UTI or UTI/OAB/UUI/no LUTS was identified.

To evaluate the hypothesis that UTIs can be the result of colonization of the urinary tract by *E. coli* strains from the gut, we examined the genome annotations for the presence of 52 genes associated with flagellar synthesis (*flg, flh*, and *fli*), flagellar rotation (*mot*), chemotactic signal transduction (*che*), and chemotactic membrane receptors (*tar, tsr, tap, trg*, and *aer*) (see section “Materials and Methods”). Twenty-four of the genomes contained all of the genes and at least one chemoreceptor, suggesting that these isolates are flagellated, motile, and chemotactic (Table 1). While all 66 genomes encode for the *flh*, *mot*, and *che* genes, the remaining 42 isolates are lacking one or more *flg* and/or *fli* genes. All 66 genomes include the *tsr, tar*, and *trg* chemoreceptors. UMB0276 is the only genome lacking the *aer* chemoreceptor and 36 lack the *tap* chemoreceptor (Supplementary Table 4).

**TABLE 1:** Number of genomes encoding for all *flg, flh, fli, mot*, and *che* genes, as well as at least one chemoreceptor.

Symptom group	# Genomes encoding for all flagellar and chemotaxis genes
no LUTS (*n* = 6)	1
OAB (*n* = 5)	0
UUI (*n* = 13)	5
UTI (*n* = 42)	18

Next, we considered pathogenicity relative to antibiotic resistance. Antibiotic resistance was predicted from the genome sequences (Supplementary Table 5). Genes associated with resistances to aminoglycosides, beta-lactams, macrolides, phenicol, quinolone, rifampicin, sulfonamides, tetracyclines, and trimethoprims were detected amongst the isolates. Most (85%) of these genes were chromosomal-mediated; only 21 strains carried plasmids encoding for an antibiotic resistance gene (Supplementary Table 5). While only UTI isolates encoded genes associated with phenicol (*n* = 2) and rifampicin (*n* = 1), all other resistances were observed in the genomes from both UTI and non-UTI isolates. Next, five antibiotics routinely prescribed for UTIs were tested: fosfomycin, ciprofloxacin, amoxicillin with clavulanic acid, sulfamethoxazole-trimethoprim, and cefpodoxime. Concurring with the genomic analysis, all of the isolates were sensitive to fosfomycin (Table 2). However, as indicated in Supplementary Table 6, computational predictions and experimental results were often discordant, e.g., resistance to ciprofloxacin was observed for 16 isolates even though quinolone resistance was only predicted for three isolates. Four of the isolates [UMB0731 (OAB) and UMB1193 (UTI), UMB1335 (UTI), and UMB1348 (UTI)] were sensitive to all five antibiotics tested. Statistical analysis found that sensitivity varied between non-UTI and UTI isolates by drug (*p* = 0.0086). Non-UTI isolates were more resistant to amoxicillin than UTI isolates and more sensitive to sulfamethoxazole-trimethoprim than UTI isolates. However, the variation in sensitivity between non-UTI and UTI isolates or amongst no LUTS, OAB, and UUI isolates was not statistically significant (*p* = 0.9682 and 0.8313, respectively).

**TABLE 2:** Percentage of *E. coli* isolates resistant or sensitive to the antibiotics fosfomycin (FOF), ciprofloxacin (CIP), amoxicillin with clavulanic acid (AMC), sulfamethoxazole-trimethoprim (SXT), and cefpodoxime (CPD).

	Symptom group	Antibiotic				
		
		FOF	CIP	AMC	SXT	CPD
Resistant	no LUTS (*n* = 6)	0	67	0	50	17
	OAB (*n* = 5)	0	40	0	60	60
	UUI (*n* = 13)	0	15	46	62	15
	UTI (*n* = 42)	0	19	5	45	26
Sensitive	no LUTS (*n* = 6)	100	33	17	17	83
	OAB (*n* = 5)	100	40	60	20	40
	UUI (*n* = 13)	100	62	54	15	62
	UTI (*n* = 42)	93	50	55	31	45

As prior studies have attributed the gastrointestinal (GI) tract as the source of UPEC strains, we next sought to compare our 66 bladder isolates with all publicly available *E. coli* genomes. It is important to note that many of the genomes deposited in GenBank were isolated either from the GI tract or from urine collected by an unspecified method during a UTI. The bladder isolates aligned to *E. coli* genomes in NCBI’s nr/nt database with 83–98% query coverage (Supplementary Table 7). These hits included isolates from urine (*n* = 24) and stool (*n* = 17) of humans, canines, cattle, mice, birds, and pigs (Table 3). Two isolates, UMB0149 (OAB) and UMB2019 (UUI), exhibited greatest similarity to isolates from forest soil and hospital wastewater, respectively (Supplementary Table 7).

**TABLE 3:** Isolation source of genomes most similar to the bladder *E. coli* isolates.

Symptom group		no LUTS (*n* = 6)	OAB (*n* = 5)	UUI (*n* = 13)	UTI (*n* = 42)
Human	Urine	1	1	2	9
	Stool	1	0	2	7
	CSF	1	0	0	4
	Blood	2	1	0	1
	Unknown	0	1	3	2
Other Species	Urine	0	1	3	7
	Stool	1	0	0	6
	Other	0	0	1	0
Hospital wastewater		0	1	0	0
Soil		0	0	1	0
Unknown		0	0	1	6

**FIGURE 3:**
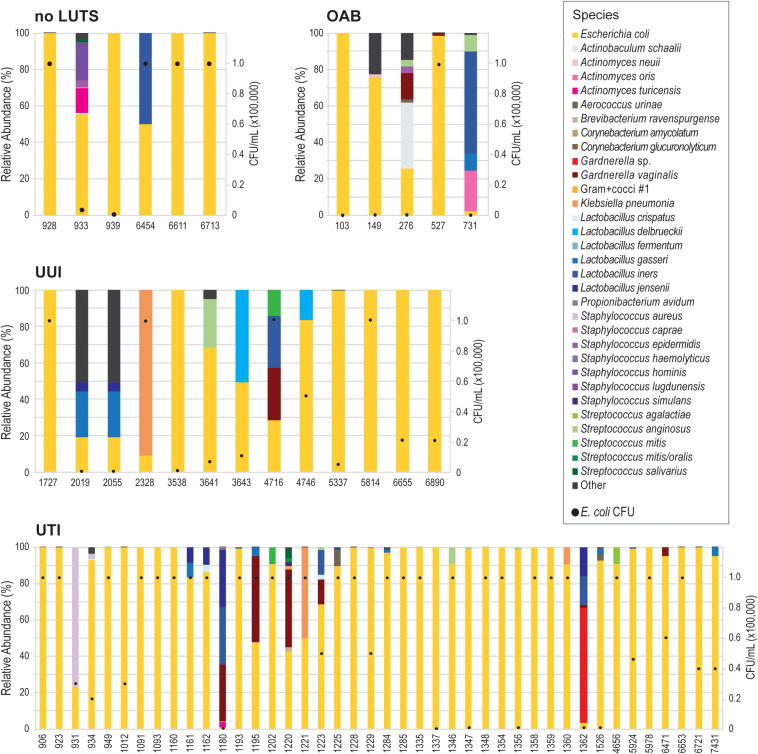
Urobiomes from which *E. coli* was isolated. Each bar represents the relative abundance of microorganisms within the urobiome, color coded by species, for the individual patient. The black circles on each stacked bar show the abundance of *E. coli* in the urobiome. Urobiomes are presented according to the symptom group.

Because our results suggest that the *E. coli* genome itself does not correspond with UTI symptoms, we next considered the *E. coli* isolates as a member of the bladder community. During the isolation of each these *E. coli* isolates, other bacterial taxa of the urobiome were also identified (Supplementary Table 8). As Figure 3 shows, several different species were found within the urobiomes of these women. Fifteen womens’ urobiomes included only *E. coli*; only 9 of these were from UTI patients. *E. coli* was the predominant member or was present in high abundance within 18 (75%) and 9 (37.5%), respectively, of the urobiomes of women without UTI symptoms. Within the UTI cohort, the urobiomes of 6 women included species from the genera *Staphylococcus*, *Lactobacillus*, *Klebsiella*, and *Gardnerella* that were in greater abundance than *E. coli*. To improve the statistical robustness of our analyses, we supplemented our urobiome community data, including CFU/mL counts for all constituents, for the 64 participants from which the 66 *E. coli* genomes were collected with an additional 135 participant data sets for a total of 109 UTI participants and 90 non-UTI no LUTS participants. While we found that *E. coli* presence was highly significant (*p*-value = 1.39E-09), the effect was very small. Our model predicted that, for a unit increase in *E. coli*, the expected odds of UTI increases by 0.003%. Thus, the presence of *E. coli* alone is not sufficient to distinguish between the urobiomes of individuals with UTI and those with no LUTS.

## Discussion

As prior studies have shown, *E. coli* can be found in the bladders of women with or without UTI symptoms (Price et al., 2016, 2020; Thomas-White et al., 2018b). Furthermore, it can be found in abundance in women without UTI symptoms [(Price et al., 2016); see also review (Ferroni and Taylor, 2015), shown also in this study (Supplementary Table 1)]. The 66 *E. coli* genomes sequenced and analyzed here include isolates obtained from women with or without UTI symptoms, as well as women with other LUTS. To our knowledge, this is the first study that has investigated *E. coli* genomes from the urobiome of women with either OAB or UUI. Prior comparative genomic studies have focused on *E. coli* isolates from individuals with or without UTI symptoms only (Rasko et al., 2008; Moriel et al., 2016, see also review; Lo et al., 2017). We also investigated the genomes of *E. coli* isolates from women without LUTS, a scenario previously called asymptomatic bacteriuria (Rubin et al., 1992; Nicolle, 2003; Nicolle et al., 2019). A recent study of 224 adult women without LUTS showed that *E. coli* is relatively uncommon in this population, but it can be present and can be the predominant taxon especially in older women (Price et al., 2020). As Figures 1, 2 show, the phylogenetic history and genomic content of *E. coli* isolates from women with or without UTI does not correspond with the participant’s UTI status. While the isolates from women with no LUTS are only found within the B2 phylotype clade, our sample size is small. We expect with an increased sample size that isolates would represent other phylotypes as isolates from the non-UTI group, which includes no LUTS, OAB, and UUI, were found in both clades of the trees.

Of the 66 isolates sequenced here, 3 were from the same individual, a woman with recurrent UTIs. The three isolates from this woman, UMB6653, UMB6721, and UMB7431, were most similar to each other in the core genome phylogeny and gene content. UMB6653 and UMB6721 are identical, according to their core gene sequences, suggesting that the same strain is responsible for the woman’s reinfection. This reinfection could be the result of persistence within the bladder over time, potentially through intracellular reservoirs, as has previously been observed within recurrent UTI patients (Mysorekar and Hultgren, 2006; Thänert et al., 2019). Our gene content analysis (Figure 2) provides evidence that this strain has been evolving over time through gene acquisition/loss; 69 genes were present in only one isolate, whereas 86 genes were present in only two of the three isolates.

Focusing our investigation of these genomes on characteristics advantageous to infection and persistence, our analysis of the accessory genome and virulence factors once again showed no distinction between those traits and symptom status. There was no gene frequently present (≥80%) in the UTI genomes that was not present in the non-UTI genomes. Prior investigations of the *E. coli* accessory genome have likewise found that symptom status and even source (urinary versus fecal) cannot reliably be distinguished by the accessory genome (Nielsen et al., 2017). Isolates from so-called asymptomatic bacteriuric individuals contain virulence factors despite the lack of symptoms (Yun et al., 2014). Here, we also have examined the genomes of *E. coli* isolates that represent a small population and/or fraction of the entire urobiome community. No distinction in virulence factors was observed between *E. coli* isolates from urobiomes in which *E. coli* was prevalent (≥100,000 CFU/ml) or dominant and those for which it was not. Together, this suggests that *E. coli* strains within the bladder have the potential for virulence, although they may not presently cause UTI symptoms.

While the *E. coli* genomes suggested that many of our isolates were resistant to antibiotics, these predictions were not significantly different between symptom states for the 9 classes of antibiotics considered (Supplementary Table 5). From our subsequent testing of 5 antibiotics, distinction between antibiotic resistance of isolates from women with or without UTI symptoms was not statistically significant. Bioinformatic predictions showed their limitations here. For example, more strains were resistant to ciprofloxacin and sulfamethoxazole-trimethoprim (16 and 32, respectively) than predicted (3 and 26, respectively). Neither the frequency of resistance predicted or observed within the no LUTS, OAB, and UUI cohorts suggests that *E. coli* strains may persist as a minor constituent of the normal bladder flora after antibiotic treatment because of acquired resistance.

There is evidence that the gut of individuals can be a reservoir for *E. coli* and thus a source of UTI (Yamamoto et al., 1997; Larsen et al., 2014). Without paired stool samples for the isolates investigated here, we cannot directly determine the source of the *E. coli* isolates. As a proxy, we compared the genomes of these isolates to publicly available genome sequences for which there are currently >19,000 from a wide variety of sources. While the most hits (36%) were to isolates from urine (Table 3), these only constitute ∼6.5% of the publicly available genomes. A substantial number of hits to isolates from stool were also observed, 10 to human stool samples and 7 to stool samples from other animals. Colonization of the urinary tract by a gut bacterium necessitates motility. However, only 24 of our isolates encode for all of the flagellar and motility genes (Table 1). For example, nine isolates lack *fliC*, which is required for the production of flagella (Lane et al., 2005). Two other isolates do not encode for *flgM*, a situation that hinders motility (Lane et al., 2005). Our investigation into these flagella-related genes suggest limited if any motility. However, as paired studies have shown, there is more than one way a UPEC strain can cause UTI recurrence. In addition to an external source, UPEC strains may persist within the urinary tract (Thänert et al., 2019).

Here, we found that neither *E. coli* presence nor *E. coli* genomic variation can predict UTI symptoms. Our analysis of urobiome compositions found that *E. coli* was a weak predictor of UTI status. Prior comparisons of urobiomes from women with or without UTI symptoms identified several differences in bacteria abundances including *Aerococcus urinae*, *Klebsiella pneumoniae*, *Enterococcus faecalis*, *E. coli*, *Staphylococcus aureus*, *Streptococcus agalactiae*, and *Streptococcus anginosus* (Price et al., 2016). These taxa include several emerging uropathogens that have increasingly been associated with UTIs (see review; Kline and Lewis, 2016). Furthermore, UTI diagnosis based upon *E. coli* abundance alone ignores recent evidence that UTI symptoms can be the result of more than one bacterial species, also known as a polymicrobial infection (see review; Kline and Lewis, 2016).

Because *E. coli* presence, abundance, and genomic content are weak predictors of UTI status, we hypothesize that UTI symptoms are more likely the result of multiple factors, including the urobiome composition. The urobiome appears to protect against post-treatment UTI (Brubaker et al., 2014; Thomas-White et al., 2018b). For instance, we previously observed that *Lactobacillus crispatus* is frequently in the urobiomes of women with no LUTS (Pearce et al., 2014). Prior studies have found that Lactobacilli can inhibit the growth of *E. coli* (Atassi et al., 2006, 2019; Cadieux et al., 2009; Hudson et al., 2020). In contrast, *E. faecalis* can promote *E. coli* growth under conditions for which it would otherwise not be able to thrive (Keogh et al., 2016). It is likely that other interactions between members of the urobiome could inhibit or contribute to symptoms. Metagenomic sequencing of “clean catch” urine specimens from individuals with or without UTI symptoms has identified urobiome compositions associated with UTIs, often dominated by Proteobacteria (Moustafa et al., 2018). These compositions require further investigation to identify constituents contributing to symptoms. In addition to microbes, other factors must also be considered, e.g., urine components (Forsyth et al., 2018; Reitzer and Zimmern, 2019) and the human host’s genetics (Ragnarsdóttir et al., 2011; Adebayo et al., 2020). Transcriptomic and proteomic studies have already begun to identify transcript and protein level changes associated with infection (Bielecki et al., 2014; Subashchandrabose et al., 2014; Sintsova et al., 2019; see also review; Lo et al., 2017). With the aid of these other ‘omic technologies, the likely multiple causes of UTI symptoms can be determined, be it a single bacterial taxon or a particular microbial community.

The *E. coli* genomes, including the presence of virulence factors and antibiotic resistance genes, from the urobiomes of women with or without UTIs cannot be distinguished. Furthermore, the *E. coli* genomes found within the urobiomes of women with OAB and UUI, which have not been previously studied, do not differ from *E. coli* genomes of isolates from women with UTI symptoms. As our analysis shows, *E. coli*’s ability to dominate the community and cause symptoms cannot be associated with a gene or set of genes. While UTI can be the result of colonization of *E. coli*, it is likely that UTI symptoms can be caused by multiple different bacteria. Recent evidence supports this hypothesis. The growing evidence of *E. coli* in the urobiomes of women without UTI symptoms suggests that *E. coli* may be a persistent member of the urinary community. This suggests that other constituents of the urobiome may directly or indirectly contribute to the development of UTI symptoms. Future investigations of the etiology of UTI symptoms should consider the entire urobiome.

## Data Availability Statement

The datasets presented in this study can be found in online repositories. The names of the repository/repositories and accession number(s) can be found at: https://www.ncbi.nlm.nih.gov/, GCF_003885035, GCF_003885055, GCF_003885095, GCF_003885125, GCF_003885145, GCF_003885155, GCF_003 885195, GCF_003885215, GCF_003885225, GCF_003885245, GCF_003885255, GCF_003885295, GCF_003885305, GCF_0038 85875, GCF_003885915, GCF_003885965, GCF_003885995, GC F_003886005, GCF_003886015, GCF_003886035, GCF_003886 045, GCF_003886095, GCF_003886105, GCF_003886115, GC F_003886135, GCF_003886175, GCF_003886185, GCF_00388 6195, GCF_003886225, GCF_003886245, GCF_003886275, GC F_003886285, GCF_003886295, GCF_003886325, GCF_00388 6345, GCF_003886375, GCF_003886385, GCF_003886395, GC F_003886435, GCF_003886445, GCF_003886455, GCF_003886 495, GCF_003886515, GCF_003886535, GCF_003886545, GC F_003886565, GCF_003886615, GCF_003886635, GCF_00388 6655, GCF_003886675, GCF_003886695, GCF_003886735, GC F_003892355, GCF_003892375, GCF_003892435, GCF_00389 2445, GCF_003892455, GCF_003892475, GCF_003892485, GC F_003892535, GCF_003892545, GCF_003892555, GCF_00389 2595, GCF_003892605, GCF_003892635, and GCF_003892645.

## Author Contributions

AGa and CP conceived of the experiment and drafted the manuscript. AGa, TM-E, AE, ZM, AS, AGe, JWS, and SB performed the data analyses. AGa, TM-E, AE, BB, GJ, RM, LM, MB, and NS assisted with experimental assays. CM assisted with writing and editing of the manuscript. AJW provided the samples and assisted with editing of the manuscript. All other authors assisted with the DNA extractions and genome assemblies as part of Loyola University Chicago’s Bacterial Genomics course. All authors contributed to the article and approved the submitted version.

## Conflict of Interest

The authors declare that the research was conducted in the absence of any commercial or financial relationships that could be construed as a potential conflict of interest

## Footnotes

1 https://github.com/najoshi/sickle

2 https://sourceforge.net/projects/bbmap/

3 www.scipy.org

4 https://www.R-project.org/
