# Supplementary material for: Genomic Survey of E. coli From the Bladders of Women With and Without Lower Urinary Tract Symptoms
Source: Front Microbiol. 2020 Sep 4;11:2094. doi: 10.3389/fmicb.2020.02094 (PMC7500147; doi:10.3389/fmicb.2020.02094)
Supplement: Supplementary file 1 [file Table_1.DOCX]

**Supplemental Table 1. *E. coli* isolate information.**

| **Strain** | **Serotype** | **Phylotype** | **Participant Symptom** | **CFU in Urine** | **% of Total Microbiota CFU** |
| --- | --- | --- | --- | --- | --- |
| 103 | O1:H6 | F | OAB | 10 | 100.00% |
| 149 | O86:H30 | A | OAB | 500 | 73.53% |
| 276 | O6:H1 | B2_3_ | OAB | 140 | 25.45% |
| 527 | O11:H4 | A | OAB | ≥100,000 | 98.44% |
| 731 | O6:H31 | B2_3_ | OAB | 20 | 2.25% |
| 906 | O25:H4 | B2_3_ | UTI | ≥100,000 | 99.89% |
| 923 | O8:H49 | B1 | UTI | ≥100,000 | 99.96% |
| 928 | O75:H5 | B2_3_ | no LUTS | ≥100,000 | 99.96% |
| 931 | O17/44:H18 | D | UTI | 30,000 | 23.00% |
| 933 | O21:H5 | B2_2_ | no LUTS | 4,000 | 55.40% |
| 934 | O11:H16 | D | UTI | 20,000 | 92.98% |
| 939 | O6:H1 | B2_3_ | no LUTS | 1,000 | 100.00% |
| 949 | O17/44:H45 | D | UTI | ≥100,000 | 99.88% |
| 1012 | O16:H5 | B2_3_ | UTI | 30,000 | 99.96% |
| 1091 | O16:H5 | B2_3_ | UTI | ≥100,000 | 100.00% |
| 1093 | O17/77:H18 | D | UTI | ≥100,000 | 100.00% |
| 1160 | O75:H5 | B2_3_ | UTI | ≥100,000 | 100.00% |
| 1161 | O25:H4 | B2_3_ | UTI | ≥100,000 | 83.33% |
| 1162 | O2:H6 | B2_3_ | UTI | ≥100,000 | 86.21% |
| 1180 | O21:H21 | B1 | UTI | 20 | 0.01% |
| 1193 | O4:H5 | B2_2_ | UTI | ≥100,000 | 99.21% |
| 1195 | O13:H4 | B2_3_ | UTI | ≥100,000 | 47.37% |
| 1202 | O8:H10 | B2_3_ | UTI | ≥100,000 | 90.90% |
| 1220 | O6:H31 | B2_3_ | UTI | ≥100,000 | 42.72% |
| 1221 | O2:H6 | B2 | UTI | ≥100,000 | 49.99% |
| 1223 | O17/44:H18 | D | UTI | 50,000 | 68.00% |
| 1225 | H34; No O | D | UTI | ≥100,000 | 89.49% |
| 1228 | O25:H4 | B2 | UTI | ≥100,000 | 99.98% |
| 1229 | O4:H5 | B2_2_ | UTI | 50,000 | 99.80% |
| 1284 | O25:H4 | B2_3_ | UTI | ≥100,000 | 96.92% |
| 1285 | O13:H4 | B2_3_ | UTI | ≥100,000 | 100.00% |
| 1335 | O2:H18 | D | UTI | ≥100,000 | 99.98% |
| 1337 | O50/2:H18 | D | UTI | 10 | 100.00% |
| 1346 | O166:H15 | D | UTI | ≥100,000 | 90.91% |
| 1347 | O166:H15 | D | UTI | 1,000 | 99.00% |
| 1348 | O75:H5 | B2_3_ | UTI | ≥100,000 | 99.00% |
| 1354 | O166:H15 | D | UTI | ≥100,000 | 99.90% |
| 1356 | H15; No O | D | UTI | 1,000 | 99.00% |
| 1358 | O147:H21 | A | UTI | ≥100,000 | 100.00% |
| 1359 | H15; No O | D | UTI | ≥100,000 | 100.00% |
| 1360 | O6:H1 | B2_3_ | UTI | ≥100,000 | 90.91% |
| 1362 | H7; No O | B1 | UTI | 1,000 | 3.17% |
| 1526 | O6:H31 | B2_3_ | UTI | 1,000 | 92.72% |
| 1727 | O17/44: H18 | D | UUI | ≥100,000 | 100.00% |
| 2019 | O19:H4 | A | UUI | 38 | 18.27% |
| 2055 | O59:H23 | B1 | UUI | 38 | 18.27% |
| 2328 | O17/77:H31 | A | UUI | ≥100,000 | 50.00% |
| 3538 | O25:H4 | B2_3_ | UUI | 150 | 100.00% |
| 3641 | O17/44:H18 | D | UUI | 6,416 | 68.35% |
| 3643 | O6:H31 | B2_3_ | UUI | 9,714 | 49.28% |
| 4656 | O1:H7 | B2_3_ | UTI | ≥100,000 | 98.74% |
| 4716 | O22:H1 | B2_3_ | UUI | ≥100,000 | 28.57% |
| 4746 | H1; No O | B2_3_ | UUI | 50,000 | 83.33% |
| 5337 | H15; No O | D | UUI | 4,667 | 99.57% |
| 5814 | O6:H31 | B2_3_ | UUI | ≥100,000 | 100.00% |
| 5924 | O75:H5 | B2_3_ | UTI | 46,000 | 99.24% |
| 5978 | O75:H5 | B2_3_ | UTI | ≥100,000 | 100.00% |
| 6454 | O2:H7 | B2_3_ | no LUTS | ≥100,000 | 50.00% |
| 6471 | O7:H15 | D | UTI | 60,400 | 95.25% |
| 6611 | O18:H7 | B2_3_ | no LUTS | ≥100,000 | 100.00% |
| 6653 | O11:H18 | D | UTI | ≥100,000 | 99.96% |
| 6655 | O75:H31 | B2_3_ | UUI | 20,571 | 99.90% |
| 6713 | O2:H7 | B2_3_ | no LUTS | ≥100,000 | 99.97% |
| 6721 | O11:H18 | D | UTI | 40,000 | 99.88% |
| 6890 | H31; No O | B2_3_ | UUI | 20,571 | 99.90% |
| 7431 | O11:H18 | D | UTI | 40,000 | 95.15% |

Symptom abbreviations: UTI = urinary tract infection; OAB = overactive bladder symptoms; UUI = urgency urinary incontinence; and no LUTS = no lower urinary tract symptoms.
