# Supplementary material for: Genomic Survey of E. coli From the Bladders of Women With and Without Lower Urinary Tract Symptoms
Source: Front Microbiol. 2020 Sep 4;11:2094. doi: 10.3389/fmicb.2020.02094 (PMC7500147; doi:10.3389/fmicb.2020.02094)
Supplement: Supplementary file 2 [file Table_2.DOCX]

**Supplemental Table 2. Genome assembly statistics.**

| **Strain** | **RefSeq Assembly Accession #** | **SRA Accession #** | **# contigs** | **#bp** | **coverage** | **# replicon sequences** |
| --- | --- | --- | --- | --- | --- | --- |
| 103 | GCF_003892645 | SRR7534319 | 76 | 5,273,431 | 102.68 | 5*^a,j,k,r,ab^* |
| 149 | GCF_003892555 | SRR7534320 | 160 | 4,986,713 | 106.56 | 1*^aa^* |
| 276 | GCF_003892545 | SRR7534321 | 76 | 5,149,559 | 103.74 | 0 |
| 527 | GCF_003892535 | SRR7534322 | 148 | 5,298,334 | 96.2 | 2*^g,o^* |
| 731 | GCF_003892485 | SRR7534279 | 45 | 5,117,715 | 106.67 | 3*^b,b,i^* |
| 906 | GCF_003886695 | SRR7534316 | 64 | 5,154,550 | 80.99 | 5*^a,b,j,k,r^* |
| 923 | GCF_003892635 | SRR7534282 | 41 | 4,804,299 | 97.61 | 1*^ab^* |
| 928 | GCF_003892445 | SRR7534318 | 64 | 5,143,148 | 93.4 | 6*^a,b,g,j,k,x^* |
| 931 | GCF_003886495 | SRR7534313 | 86 | 5,589,631 | 98.72 | 5*^j,k,r,w,z^* |
| 933 | GCF_003886675 | SRR7534314 | 91 | 5,173,216 | 111.29 | 4*^b,i,k,o^* |
| 934 | GCF_003892475 | SRR7534305 | 117 | 5,307,043 | 67.53 | 3*^j,k,r^* |
| 939 | GCF_003885295 | SRR7534289 | 80 | 5,136,252 | 67.71 | 2*^d,e^* |
| 949 | GCF_003892435 | SRR7534306 | 92 | 5,286,823 | 111.99 | 5*^b,j,k,o,o^* |
| 1012 | GCF_003886455 | SRR7534307 | 86 | 4,904,166 | 95.45 | 5*^b,j,l,q,r^* |
| 1091 | GCF_003886445 | SRR7534308 | 85 | 5,366,077 | 90.92 | 6*^b,f,k,o,p,q^* |
| 1093 | GCF_003885215 | SRR7534290 | 99 | 5,448,718 | 95.94 | 4*^a,k,p,x^* |
| 1160 | GCF_003892605 | SRR7534309 | 69 | 5,315,769 | 91.92 | 3*^b,k,o^* |
| 1161 | GCF_003885195 | SRR7534291 | 94 | 5,183,853 | 58.53 | 5*^b,j,k,q,r^* |
| 1162 | GCF_003892455 | SRR7534310 | 79 | 5,317,917 | 71.32 | 3*^k,o,ab^* |
| 1180 | GCF_003892375 | SRR7534311 | 52 | 4,692,379 | 98.38 | 1*^c^* |
| 1193 | GCF_003892595 | SRR7534312 | 61 | 5,122,540 | 79.91 | 3*^b,k,o^* |
| 1195 | GCF_003886435 | SRR7534297 | 50 | 5,099,255 | 85.71 | 3*^i,k,o^* |
| 1202 | GCF_003886395 | SRR7534298 | 40 | 4,871,500 | 75.16 | 2*^k,o^* |
| 1220 | GCF_003886385 | SRR7534271 | 59 | 5,003,938 | 79.7 | 0 |
| 1221 | GCF_003885055 | SRR8185535 | 64 | 5,320,813 | 91.6 | 3*^k,n,ab^* |
| 1223 | GCF_003886375 | SRR7534270 | 84 | 5,313,703 | 87.31 | 4*^b,j,k,r^* |
| 1225 | GCF_003886735 | SRR7534273 | 56 | 4,744,454 | 60.45 | 0 |
| 1228 | GCF_003886655 | SRR7534272 | 67 | 5,102,328 | 77.25 | 1*^y^* |
| 1229 | GCF_003886345 | SRR7534267 | 84 | 5,119,228 | 49.04 | 3*^b,k,o^* |
| 1284 | GCF_003892355 | SRR7534266 | 80 | 5,171,040 | 54.66 | 3*^j,o,z^* |
| 1285 | GCF_003886635 | SRR7534269 | 55 | 4,899,007 | 55.71 | 2*^k,o^* |
| 1335 | GCF_003886615 | SRR7534268 | 98 | 5,388,660 | 64.77 | 4*^b,k,p,u^* |
| 1337 | GCF_003886325 | SRR7534277 | 77 | 5,365,127 | 59.85 | 4*^b,k,p,u^* |
| 1346 | GCF_003886295 | SRR7534276 | 41 | 4,837,249 | 99.88 | 1*^p^* |
| 1347 | GCF_003886285 | SRR7534303 | 46 | 4,836,273 | 133.95 | 1*^p^* |
| 1348 | GCF_003886275 | SRR7534304 | 64 | 5,247,988 | 107.47 | 4*^b,k,p,y^* |
| 1354 | GCF_003886225 | SRR7534301 | 39 | 4,776,999 | 99.71 | 0 |
| 1356 | GCF_003886245 | SRR7534302 | 46 | 4,770,454 | 130.59 | 0 |
| 1358 | GCF_003886195 | SRR7534299 | 95 | 4,954,546 | 95.21 | 2*^s,t^* |
| 1359 | GCF_003886185 | SRR7534300 | 56 | 4,768,910 | 78.91 | 0 |
| 1360 | GCF_003886565 | SRR7534285 | 65 | 5,066,683 | 83.35 | 3*^b,k,p^* |
| 1362 | GCF_003886175 | SRR7534274 | 110 | 4,931,742 | 89.3 | 2*^a,k^* |
| 1526 | GCF_003886105 | SRR7534315 | 54 | 4,985,292 | 79.96 | 1*^x^* |
| 1727 | GCF_003886135 | SRR7534325 | 112 | 5,424,027 | 66.47 | 5*^i,k,o,o,z^* |
| 2019 | GCF_003886115 | SRR7534280 | 161 | 5,031,974 | 65.64 | 4*^c,g,v,aa^* |
| 2055 | GCF_003886095 | SRR7534278 | 52 | 4,893,115 | 83.43 | 2*^g,v^* |
| 2328 | GCF_003886545 | SRR7534293 | 92 | 4,602,187 | 72.99 | 1*^g^* |
| 3538 | GCF_003886535 | SRR7534286 | 78 | 5,152,159 | 80.67 | 5*^a,j,k,r,u^* |
| 3641 | GCF_003885305 | SRR7534292 | 91 | 5,150,305 | 116.13 | 3*^j,k,r^* |
| 3643 | GCF_003885095 | SRR8185536 | 54 | 5,284,698 | 75.96 | 2*^g,o^* |
| 4656 | GCF_003886515 | SRR7534284 | 54 | 5,189,782 | 137.14 | 4*^b,k,o,p^* |
| 4716 | GCF_003885995 | SRR7534287 | 68 | 5,323,152 | 92.54 | 4*^b,k,p,aa^* |
| 4746 | GCF_003886045 | SRR7534283 | 63 | 5,051,634 | 104.84 | 0 |
| 5337 | GCF_003886035 | SRR7534281 | 107 | 4,823,947 | 24.92 | 1*^p^* |
| 5814 | GCF_003886015 | SRR7534275 | 41 | 4,985,835 | 89.47 | 1*^x^* |
| 5924 | GCF_003886005 | SRR7534324 | 72 | 5,024,439 | 84.69 | 4*^b,b,j,k^* |
| 5978 | GCF_003885915 | SRR7534323 | 60 | 5,086,444 | 92.1 | 5*^a,b,j,k,x^* |
| 6454 | GCF_003885245 | SRR7534294 | 33 | 5,108,808 | 126.66 | 3*^b,k,p^* |
| 6471 | GCF_003885155 | SRR7534327 | 44 | 5,008,049 | 89.74 | 3*^b,k,p^* |
| 6611 | GCF_003885875 | SRR7534295 | 73 | 5,153,766 | 112.32 | 3*^b,k,o^* |
| 6653 | GCF_003885965 | SRR7534296, SRR8182356 | 88 | 5,355,464 | 78.32 | 5*^b,i,k,p,z^* |
| 6655 | GCF_003885255 | SRR7534326 | 43 | 5,119,947 | 55.6 | 3*^b,h,m^* |
| 6713 | GCF_003885145 | SRR7534329 | 46 | 5,169,541 | 87.26 | 4*^b,i,k,p^* |
| 6721 | GCF_003885125 | SRR7534328, SRR8182355 | 74 | 5,314,438 | 78.2 | 5*^b,i,k,p,z^* |
| 6890 | GCF_003885035 | SRR7534331 | 45 | 5,100,795 | 63.12 | 3*^b,h,m^* |
| 7431 | GCF_003885225 | SRR7534330, SRR8182357 | 71 | 5,313,988 | 75.49 | 4*^i,k,p,z^* |

**Plasmid:** *a*: Col(BS512); *b*: Col156; *c*: Col440I; *d*: Col(MG828); *e*: ColRNAI; *f*: ColpVC; *g*: Incl1-I(Gamma); *h*: Incl2(Delta); *i*: IncB/O/K/Z; *j*: IncFIA; *k*: IncFIB(AP001918); *l*: IncFIB(pB171); *m*: IncFIB(H89-PhagePlasmid); *n*: IncFIC(FII); *o*: IncFII; *p*: IncFII(29); *q*: IncFII(pCoo); *r*: IncFII(pRSB107); *s*: IncHI1A; *t*: IncHI1B(R27); *u*: IncN; *v*: IncN3; *w*: IncP1; *x*: IncQ1; *y*: IncX1; *z*: IncX4; *aa*: IncY; *ab*: p0111.
