# Supplementary material for: Genomic Survey of E. coli From the Bladders of Women With and Without Lower Urinary Tract Symptoms
Source: Front Microbiol. 2020 Sep 4;11:2094. doi: 10.3389/fmicb.2020.02094 (PMC7500147; doi:10.3389/fmicb.2020.02094)
Supplement: Supplementary file 3 [file Table_3.DOCX]

**Supplemental Table 3. Virulence factors (VF) identified for genomes by participant symptom group.**

| **Vfclass** | **Virulence factors** | **Related genes** |  | **Non-UTI (n=24)** | | |
| --- | --- | --- | --- | --- | --- | --- |
|  |  |  | **UTI (n=42)** | **no LUTS (n=6)** | **OAB (n=5)** | **UUI (n=13)** |
| Adherence | AAF/II fimbriae | *aafC* | 3 | 1 | 0 | 0 |
|  |  | *agg3C* | 0 | 0 | 0 | 1 |
|  |  | *agg3D* | 0 | 0 | 0 | 1 |
|  | Afimbrial adhesin AFA-I | *afaA* | 3 | 1 | 0 | 0 |
|  |  | *afaB* | 4 | 2 | 0 | 0 |
|  |  | *afaC* | 1 | 2 | 0 | 1 |
|  |  | *afaD* | 3 | 1 | 0 | 0 |
|  |  | *draP* | 3 | 1 | 0 | 0 |
|  | CFA/I fimbriae | *cfaA* | 8 | 0 | 3 | 6 |
|  |  | *cfaB* | 8 | 0 | 3 | 6 |
|  |  | *cfaC* | 8 | 0 | 3 | 6 |
|  |  | *cfaD/cfaE* | 8 | 0 | 3 | 6 |
|  | Dispersin | *aap* | 1 | 0 | 0 | 1 |
|  | E. coli common pilus (ECP) | *ecpA* | 41 | 6 | 4 | 11 |
|  |  | *ecpB* | 41 | 6 | 4 | 11 |
|  |  | *ecpC* | 41 | 6 | 4 | 11 |
|  |  | *ecpD* | 41 | 6 | 4 | 11 |
|  |  | *ecpE* | 41 | 6 | 4 | 11 |
|  |  | *ecpR* | 40 | 6 | 4 | 11 |
|  | E.coli laminin-binding fimbriae (ELF) | *elfA* | 13 | 0 | 3 | 4 |
|  |  | *elfC* | 12 | 0 | 3 | 4 |
|  |  | *elfD* | 13 | 0 | 3 | 4 |
|  |  | *elfG* | 13 | 0 | 2 | 3 |
|  | EaeH | *eaeH* | 39 | 6 | 4 | 10 |
|  | EtpA | *etpA* | 1 | 0 | 0 | 0 |
|  | F1C fimbriae | *focA* | 2 | 1 | 2 | 5 |
|  |  | *focC* | 4 | 2 | 2 | 6 |
|  |  | *focD* | 4 | 2 | 2 | 5 |
|  |  | *focF* | 4 | 2 | 2 | 6 |
|  |  | *focG* | 0 | 1 | 1 | 1 |
|  |  | *focH* | 4 | 2 | 2 | 6 |
|  |  | *focI* | 4 | 2 | 2 | 5 |
|  | Hemorrhagic E. coli pilus (HCP) | *hcpA* | 42 | 6 | 5 | 13 |
|  |  | *hcpB* | 38 | 6 | 5 | 12 |
|  |  | *hcpC* | 42 | 6 | 4 | 13 |
|  | P fimbriae | *papA* | 22 | 3 | 3 | 5 |
|  |  | *papB* | 11 | 4 | 2 | 4 |
|  |  | *papC* | 19 | 4 | 2 | 5 |
|  |  | *papD* | 21 | 4 | 2 | 5 |
|  |  | *papE* | 16 | 4 | 3 | 5 |
|  |  | *papF* | 16 | 4 | 2 | 5 |
|  |  | *papG* | 11 | 4 | 2 | 4 |
|  |  | *papH* | 18 | 4 | 2 | 5 |
|  |  | *papI* | 26 | 6 | 3 | 6 |
|  |  | *papJ* | 10 | 3 | 2 | 4 |
|  |  | *papK* | 12 | 4 | 2 | 4 |
|  |  | *papX* | 22 | 6 | 4 | 8 |
|  | Porcine attaching-effacing associated protein | *paa* | 1 | 0 | 0 | 0 |
|  | S fimbriae | *sfaA* | 2 | 0 | 1 | 2 |
|  |  | *sfaB* | 6 | 1 | 2 | 6 |
|  |  | *sfaC* | 0 | 1 | 0 | 2 |
|  |  | *sfaS* | 4 | 1 | 1 | 5 |
|  | ToxB | *toxB* | 1 | 0 | 0 | 0 |
|  | Type 3 fimbriae (Klebsiella) | *mrkB* | 0 | 0 | 1 | 1 |
|  |  | *mrkD* | 0 | 0 | 1 | 1 |
|  |  | *-* | 0 | 0 | 1 | 1 |
|  | Type I fimbriae | *fimA* | 41 | 6 | 4 | 13 |
|  |  | *fimB* | 37 | 6 | 4 | 12 |
|  |  | *fimC* | 41 | 6 | 4 | 13 |
|  |  | *fimD* | 41 | 6 | 5 | 13 |
|  |  | *fimE* | 40 | 6 | 4 | 13 |
|  |  | *fimF* | 42 | 6 | 5 | 13 |
|  |  | *fimG* | 42 | 6 | 5 | 13 |
|  |  | *fimH* | 42 | 6 | 4 | 13 |
|  |  | *fimI* | 40 | 6 | 4 | 13 |
|  | Type I fimbriae (Klebsiella) | *-* | 2 | 0 | 0 | 0 |
|  | Type IV pili (Yersinia) | *pilQ* | 0 | 1 | 1 | 4 |
|  |  | *pilR* | 0 | 1 | 1 | 4 |
|  |  | *pilS* | 0 | 1 | 1 | 4 |
|  |  | *pilV* | 0 | 1 | 1 | 4 |
|  |  | *pilW* | 0 | 1 | 2 | 3 |
| Antiphagocytosis | Capsular polysaccharide (Vibrio) | *wbjD/wecB* | 3 | 0 | 1 | 0 |
|  |  | *wecC* | 1 | 0 | 0 | 0 |
|  | Capsule (Klebsiella) | *-* | 6 | 0 | 1 | 0 |
|  |  | *uge* | 1 | 0 | 0 | 0 |
| Autotransporter | AatA | *aatA* | 1 | 0 | 0 | 0 |
|  | Antigen 43 | *agn43* | 19 | 3 | 2 | 5 |
|  | Cah | *cah* | 17 | 4 | 1 | 4 |
|  | Contact-dependent inhibition CDI system | *cdiA* | 6 | 2 | 2 | 5 |
|  | EhaA | *ehaA* | 1 | 0 | 0 | 0 |
|  | EhaB | *ehaB* | 42 | 6 | 5 | 13 |
|  | Enteroaggregative immunoglobulin repeat protein | *air/eaeX* | 11 | 0 | 1 | 2 |
|  | EspC | *espC* | 8 | 0 | 0 | 1 |
|  | EspP | *espP* | 2 | 0 | 0 | 0 |
|  | Pic | *pic* | 1 | 1 | 1 | 2 |
|  | Sat | *sat* | 15 | 1 | 2 | 2 |
|  | Temperature-sensitive hemagglutinin | *tsh* | 13 | 1 | 0 | 1 |
|  | UpaG adhesin | *upaG/ehaG* | 29 | 4 | 2 | 13 |
|  | UpaH | *upaH* | 15 | 0 | 1 | 6 |
|  | Vacuolating autotransporter gene | *vat* | 11 | 3 | 1 | 4 |
| Colonization and Immune evasion | Capsule biosynthesis and transport (Campylobacter) | *glf* | 2 | 0 | 0 | 0 |
| Fimbrial adherence determinants | Lpf (Salmonella) | *lpfB* | 3 | 0 | 0 | 1 |
|  |  | *lpfC* | 3 | 0 | 0 | 1 |
|  |  | *lpfE* | 3 | 0 | 0 | 1 |
|  | Pef (Salmonella) | *pefC* | 0 | 1 | 0 | 0 |
|  | Pef (Salmonella) | *pefD* | 0 | 1 | 0 | 0 |
|  | Stj (Salmonella) | *stjC* | 1 | 0 | 1 | 1 |
| Immune evasion | Capsule (Acinetobacter) | *-* | 6 | 2 | 0 | 0 |
|  | Exopolysaccharide (Haemophilus) | *galE* | 5 | 0 | 0 | 3 |
|  | LOS (Campylobacter) | *-* | 4 | 2 | 0 | 0 |
|  | LPS glucosylation (Shigella) | *gtr* | 1 | 0 | 0 | 0 |
| Invasion | Invasin A (Yersinia) | *-* | 1 | 0 | 0 | 0 |
|  | Invasion of brain endothelial cells (Ibes) | *ibeA* | 2 | 1 | 0 | 0 |
|  |  | *ibeB* | 42 | 6 | 5 | 13 |
|  |  | *ibeC* | 42 | 6 | 5 | 13 |
|  | Tia/Hek | *tia* | 10 | 5 | 2 | 6 |
| Iron uptake | Aerobactin siderophore | *iucA* | 18 | 2 | 3 | 2 |
|  |  | *iucB* | 19 | 2 | 3 | 2 |
|  |  | *iucC* | 19 | 2 | 3 | 2 |
|  |  | *iucD* | 19 | 2 | 3 | 3 |
|  |  | *iutA* | 19 | 2 | 3 | 3 |
|  | Heme uptake | *chuA* | 38 | 6 | 3 | 9 |
|  |  | *chuS* | 38 | 6 | 3 | 10 |
|  |  | *chuT* | 38 | 6 | 3 | 10 |
|  |  | *chuU* | 38 | 6 | 3 | 10 |
|  |  | *chuW* | 38 | 6 | 3 | 10 |
|  |  | *chuX* | 38 | 6 | 3 | 10 |
|  |  | *chuY* | 38 | 6 | 3 | 10 |
|  | Iron-regulated element | *ireA* | 5 | 2 | 1 | 1 |
|  | Iron/manganese transport | *sitA* | 32 | 6 | 3 | 7 |
|  |  | *sitB* | 32 | 6 | 3 | 7 |
|  |  | *sitC* | 31 | 6 | 3 | 7 |
|  |  | *sitD* | 32 | 6 | 3 | 7 |
|  | Salmochelin siderophore | *iroB* | 7 | 2 | 2 | 5 |
|  |  | *iroC* | 7 | 2 | 2 | 5 |
|  |  | *iroD* | 7 | 2 | 2 | 4 |
|  |  | *iroE* | 7 | 2 | 2 | 5 |
|  |  | *iroN* | 7 | 2 | 2 | 5 |
|  | Yersiniabactin siderophore | *fyuA* | 33 | 6 | 5 | 9 |
|  |  | *irp1* | 32 | 6 | 5 | 9 |
|  |  | *irp2* | 28 | 4 | 5 | 7 |
|  |  | *ybtA* | 33 | 6 | 5 | 9 |
|  |  | *ybtE* | 33 | 6 | 5 | 9 |
|  |  | *ybtP* | 33 | 6 | 5 | 9 |
|  |  | *ybtQ* | 33 | 6 | 5 | 9 |
|  |  | *ybtS* | 33 | 5 | 5 | 9 |
|  |  | *ybtT* | 33 | 6 | 5 | 9 |
|  |  | *ybtU* | 33 | 6 | 5 | 9 |
|  |  | *ybtX* | 33 | 6 | 5 | 9 |
| Motility | Flagella (Bordetella) | *flaA* | 6 | 1 | 0 | 0 |
| Non-LEE encoded TTSS effectors | EspFu/TccP (Tir cytoskeleton coupling protein) | *espFu/tccP* | 1 | 0 | 0 | 0 |
|  | EspL1 | *espL1* | 14 | 0 | 1 | 3 |
|  | EspL4 | *espL4* | 20 | 0 | 1 | 5 |
|  | EspM2 | *espM2* | 1 | 0 | 0 | 0 |
|  | EspO1-1 | *espO1-1* | 1 | 0 | 0 | 0 |
|  | EspO1-2 | *espO1-2* | 1 | 0 | 0 | 0 |
|  | EspR1 | *espR1* | 21 | 0 | 3 | 6 |
|  | EspR4 | *espR4* | 11 | 0 | 2 | 2 |
|  | EspX1 | *espX1* | 20 | 0 | 2 | 4 |
|  | EspX2 | *espX2* | 7 | 0 | 0 | 1 |
|  | EspX4 | *espX4* | 17 | 0 | 1 | 4 |
|  | EspX5 | *espX5* | 21 | 0 | 1 | 5 |
|  | EspX6 | *espX6* | 7 | 0 | 0 | 1 |
|  | EspY1 | *espY1* | 17 | 0 | 1 | 3 |
|  | EspY2 | *espY2* | 17 | 0 | 1 | 3 |
|  | EspY3 | *espY3* | 15 | 0 | 0 | 1 |
|  | EspY4 | *espY4* | 17 | 0 | 1 | 2 |
|  | EspY5 | *espY5* | 7 | 0 | 0 | 0 |
|  | NleA | *nleA* | 1 | 0 | 0 | 0 |
|  | NleB2-1 | *nleB2-1* | 1 | 0 | 0 | 0 |
|  | NleC | *nleC* | 1 | 0 | 0 | 0 |
|  | NleF | *nleF* | 1 | 0 | 0 | 0 |
|  | NleG-1 | *nleG-1* | 1 | 0 | 0 | 0 |
|  | NleG2-2 | *nleG2-2* | 1 | 0 | 0 | 0 |
|  | NleH1-1 | *nleH1-1* | 1 | 0 | 0 | 0 |
| Others | O-antigen (Yersinia) | *cpsB* | 4 | 0 | 1 | 0 |
|  |  | *fcl* | 4 | 0 | 1 | 0 |
|  |  | *galE* | 11 | 0 | 1 | 2 |
|  |  | *-* | 7 | 0 | 1 | 2 |
|  | VirF (Shigella) | *virF* | 0 | 2 | 0 | 0 |
| Protease | IcsP (SopA) (Shigella) | *icsP/sopA* | 1 | 0 | 0 | 0 |
| Regulation | AggR | *aggR* | 1 | 0 | 0 | 0 |
| Secretion system | AAI/SCI-II T6SS | *aaiA* | 1 | 0 | 0 | 0 |
|  |  | *aaiB* | 1 | 0 | 0 | 0 |
|  |  | *aaiI* | 1 | 0 | 0 | 0 |
|  |  | *clpV/aaiP* | 1 | 0 | 0 | 0 |
|  | ABC transporter for dispersin | *aatA* | 1 | 0 | 0 | 1 |
|  |  | *aatB* | 1 | 0 | 0 | 1 |
|  |  | *aatC* | 1 | 0 | 0 | 0 |
|  |  | *aatP* | 1 | 0 | 0 | 1 |
|  | ACE T6SS | *aec11* | 9 | 5 | 1 | 4 |
|  |  | *aec14* | 9 | 5 | 1 | 4 |
|  |  | *aec15* | 32 | 3 | 4 | 9 |
|  |  | *aec16* | 31 | 5 | 3 | 6 |
|  |  | *aec17* | 32 | 5 | 3 | 7 |
|  |  | *aec18* | 32 | 5 | 3 | 7 |
|  |  | *aec19* | 32 | 5 | 3 | 7 |
|  |  | *aec22* | 31 | 5 | 3 | 7 |
|  |  | *aec23* | 31 | 5 | 3 | 7 |
|  |  | *aec24* | 31 | 5 | 3 | 7 |
|  |  | *aec25* | 31 | 5 | 3 | 7 |
|  |  | *aec26* | 31 | 5 | 3 | 7 |
|  |  | *aec27/clpV* | 31 | 5 | 3 | 7 |
|  |  | *aec28* | 31 | 5 | 3 | 7 |
|  |  | *aec29* | 31 | 5 | 3 | 7 |
|  |  | *aec30* | 31 | 5 | 2 | 7 |
|  |  | *aec31* | 32 | 5 | 3 | 8 |
|  |  | *aec32* | 33 | 5 | 3 | 8 |
|  |  | *aec7* | 19 | 5 | 1 | 6 |
|  |  | *aec8* | 19 | 4 | 0 | 6 |
|  |  | Undetermined | 31 | 5 | 3 | 7 |
|  |  | Undetermined | 9 | 5 | 1 | 4 |
|  | Hcp secretion island-1 encoded type VI secretion system (H-T6SS) (Pseudomonas) | *clpV1* | 2 | 0 | 0 | 1 |
|  |  | *-* | 2 | 0 | 0 | 1 |
|  | icm/dot type IVB locus (Yersinia) | *-* | 0 | 0 | 1 | 0 |
|  | Lvh (Legionella vir homologs) type IVA secretion system (Legionella) | *traG* | 1 | 0 | 0 | 0 |
|  |  | *trbE* | 2 | 0 | 0 | 0 |
|  |  | *virB* | 2 | 0 | 0 | 0 |
|  | SCI-I T6SS | Undetermined | 20 | 6 | 2 | 8 |
|  |  | Undetermined | 20 | 6 | 2 | 8 |
|  |  | Undetermined | 8 | 0 | 0 | 1 |
|  |  | Undetermined | 5 | 0 | 0 | 1 |
|  |  | Undetermined | 8 | 0 | 0 | 1 |
|  |  | Undetermined | 4 | 4 | 1 | 2 |
|  |  | Undetermined | 12 | 5 | 2 | 7 |
|  |  | Undetermined | 20 | 6 | 2 | 8 |
|  |  | Undetermined | 20 | 6 | 2 | 8 |
|  |  | Undetermined | 20 | 6 | 2 | 8 |
|  |  | Undetermined | 20 | 6 | 1 | 8 |
|  |  | Undetermined | 20 | 6 | 2 | 8 |
|  |  | Undetermined | 20 | 6 | 2 | 8 |
|  |  | Undetermined | 1 | 0 | 1 | 0 |
|  |  | Undetermined | 6 | 3 | 0 | 0 |
|  |  | Undetermined | 20 | 6 | 2 | 8 |
|  |  | Undetermined | 20 | 6 | 2 | 8 |
|  |  | Undetermined | 18 | 6 | 2 | 8 |
|  |  | Undetermined | 19 | 6 | 2 | 8 |
|  |  | Undetermined | 19 | 6 | 2 | 8 |
|  | T6SS (Aeromonas) | *-* | 5 | 1 | 0 | 0 |
| Serum resistance | LPS rfb locus (Klebsiella) | *-* | 3 | 0 | 0 | 1 |
| Stress adaptation | Manganese transport system (Neisseria) | *mntB* | 2 | 0 | 0 | 0 |
| Toxin | Alpha-hemolysin | *hlyA* | 10 | 2 | 2 | 5 |
|  |  | *hlyB* | 10 | 2 | 2 | 6 |
|  |  | *hlyC* | 10 | 2 | 2 | 6 |
|  |  | *hlyD* | 10 | 2 | 2 | 6 |
|  | Colicin-like Usp | *usp* | 20 | 6 | 2 | 7 |
|  | Cytolethal distending toxin | *cdtA* | 1 | 0 | 0 | 0 |
|  |  | *cdtB* | 1 | 0 | 0 | 0 |
|  |  | *cdtC* | 1 | 0 | 0 | 0 |
|  | Cytotoxic necrotizing factor 1 | *cnf1* | 5 | 2 | 1 | 4 |
|  | Enterotoxin SenB/TieB | *senB* | 20 | 5 | 1 | 2 |
|  | Hemolysin/cytolysin A | *hlyE/clyA* | 20 | 0 | 3 | 4 |

Related genes listed as “Undetermined” are labeled as such by VFDB. Those listed as “-“ do not have any label listed in VFDB; the field is blank. Symptom abbreviations: UTI = urinary tract infection; OAB = overactive bladder symptoms; UUI = urgency urinary incontinence; and no LUTS = no lower urinary tract symptoms.
