# Supplementary material for: Genomic Survey of E. coli From the Bladders of Women With and Without Lower Urinary Tract Symptoms
Source: Front Microbiol. 2020 Sep 4;11:2094. doi: 10.3389/fmicb.2020.02094 (PMC7500147; doi:10.3389/fmicb.2020.02094)
Supplement: Supplementary file 4 [file Table_4.DOCX]

**Supplemental Table 4. Strains missing one or more genes associated with flagellar synthesis.** “X” indicates that the gene is not found within the genome sequence.

| **Strain** | **Participant Symptom** | ***flgH*** | ***flgI*** | ***flgM*** | ***fliC*** | ***fliD*** | ***fliF*** | ***fliM*** | ***fliP*** | ***fliR*** |
| --- | --- | --- | --- | --- | --- | --- | --- | --- | --- | --- |
| 103 | OAB | X | X |  |  |  |  |  |  |  |
| 149 | OAB | X | X |  |  |  |  |  |  |  |
| 276 | OAB | X | X |  |  |  |  |  |  |  |
| 527 | OAB | X | X |  | X |  |  |  | X |  |
| 731 | OAB | X | X |  |  |  |  |  |  |  |
| 906 | UTI |  |  |  | X |  |  |  |  |  |
| 923 | UTI | X | X |  |  |  |  |  |  |  |
| 933 | no LUTS | X | X |  |  |  |  |  |  |  |
| 934 | UTI |  |  |  |  | X |  |  |  |  |
| 939 | no LUTS | X | X |  |  |  |  |  |  |  |
| 1012 | UTI | X | X |  |  |  |  |  |  |  |
| 1091 | UTI | X | X |  |  |  |  |  |  |  |
| 1161 | UTI |  |  |  | X |  |  |  |  |  |
| 1162 | UTI | X | X |  |  |  |  |  |  |  |
| 1180 | UTI | X | X |  | X | X |  |  |  |  |
| 1193 | UTI | X | X | X |  |  |  |  |  |  |
| 1195 | UTI | X | X |  | X |  |  |  |  |  |
| 1220 | UTI | X | X |  |  |  |  |  |  |  |
| 1221 | UTI | X | X |  |  |  |  |  |  |  |
| 1228 | UTI |  |  |  | X |  |  |  |  |  |
| 1229 | UTI | X | X | X |  |  |  |  |  |  |
| 1284 | UTI |  |  |  | X |  |  |  |  |  |
| 1285 | UTI | X | X |  | X |  |  |  |  |  |
| 1335 | UTI | X | X |  |  |  |  |  |  |  |
| 1337 | UTI | X | X |  |  |  |  |  |  |  |
| 1354 | UTI |  |  |  |  |  | X |  |  |  |
| 1358 | UTI | X | X |  |  | X |  |  |  |  |
| 1360 | UTI | X | X |  |  |  |  |  |  |  |
| 1362 | UTI | X | X |  |  |  |  |  |  |  |
| 1526 | UTI | X | X |  |  |  |  |  |  |  |
| 2019 | UUI | X | X |  |  |  |  |  |  |  |
| 3538 | UUI |  |  |  | X |  |  |  |  |  |
| 3643 | UUI | X | X |  |  |  |  |  |  |  |
| 4656 | UTI | X | X |  |  |  |  |  |  | X |
| 4716 | UUI | X | X |  |  |  |  |  |  |  |
| 4746 | UUI | X | X |  |  |  |  |  |  |  |
| 5814 | UUI | X | X |  |  |  |  |  |  |  |
| 6454 | no LUTS | X | X |  |  |  |  |  |  |  |
| 6611 | no LUTS | X | X |  |  |  |  |  |  |  |
| 6655 | UUI | X | X |  |  |  |  | X |  |  |
| 6713 | no LUTS | X | X |  |  |  |  |  |  |  |
| 6890 | UUI | X | X |  |  |  |  | X |  |  |

Symptom abbreviations: UTI = urinary tract infection; OAB = overactive bladder symptoms; UUI = urgency urinary incontinence; and no LUTS = no lower urinary tract symptoms.
