# Supplementary material for: Genomic Survey of E. coli From the Bladders of Women With and Without Lower Urinary Tract Symptoms
Source: Front Microbiol. 2020 Sep 4;11:2094. doi: 10.3389/fmicb.2020.02094 (PMC7500147; doi:10.3389/fmicb.2020.02094)
Supplement: Supplementary file 5 [file Table_5.DOCX]

**Supplemental Table 5. Antibiotic resistances predicted from the genome sequence by ResFinder.** “X” indicates that the strain includes a gene(s) associated with resistance to the listed antibiotic.

| **Strain** | **Participant Symptom** | **Aminoglycoside** | **Beta-lactam** | **Macrolide** | **Phenicol** | **Quinolone** | **Rifampicin** | **Sulphonamide** | **Tetracycline** | **Trimethoprim** |
| --- | --- | --- | --- | --- | --- | --- | --- | --- | --- | --- |
| 103 | OAB | X | X | X |  |  |  | X | X | X |
| 149 | OAB |  |  | X |  |  |  |  |  |  |
| 276 | OAB |  | X | X |  |  |  |  |  |  |
| 527 | OAB |  |  | X |  |  |  |  |  |  |
| 731 | OAB |  |  | X |  |  |  |  |  |  |
| 906 | UTI | X | X*^P^* | X |  |  |  | X | X | X |
| 923 | UTI |  |  | X |  |  |  |  |  |  |
| 928 | no LUTS | X*^P^* | X | X |  |  |  | X*^P^* | X | X |
| 931 | UTI | X | X | X |  |  |  | X | X | X |
| 933 | no LUTS | X |  | X |  |  |  | X*^P^* |  | X |
| 934 | UTI | X | X | X |  |  |  | X | X | X |
| 939 | no LUTS |  | X*^P^* | X |  |  |  |  | X |  |
| 949 | UTI | X | X*^P^* | X |  |  |  | X | X | X |
| 1012 | UTI |  | X | X |  |  |  |  |  |  |
| 1091 | UTI | X | X | X |  |  |  | X | X | X |
| 1093 | UTI |  | X | X | X |  |  | X*^P^* |  |  |
| 1160 | UTI | X*^P^* |  | X*^P^* |  |  |  | X*^P^* |  | X*^P^* |
| 1161 | UTI | X | X*^P^* | X |  | X | X | X | X | X |
| 1162 | UTI |  |  | X |  |  |  | X | X |  |
| 1180 | UTI |  |  | X |  |  |  |  |  |  |
| 1193 | UTI | X*^P^* | X | X |  |  |  | X | X |  |
| 1195 | UTI |  |  | X |  |  |  |  |  |  |
| 1202 | UTI |  |  | X |  |  |  |  |  |  |
| 1220 | UTI |  |  | X |  |  |  |  |  |  |
| 1221 | UTI |  |  | X |  |  |  | X | X |  |
| 1223 | UTI | X | X | X |  |  |  | X | X | X |
| 1225 | UTI |  |  |  |  |  |  |  |  |  |
| 1228 | UTI |  |  | X |  |  |  |  |  |  |
| 1229 | UTI | X*^P^* | X | X |  |  |  | X | X |  |
| 1284 | UTI | X | X | X | X | X |  | X | X*^P^* | X |
| 1285 | UTI |  |  | X |  |  |  |  |  |  |
| 1335 | UTI |  | X | X |  |  |  |  |  |  |
| 1337 | UTI |  | X | X |  |  |  |  |  |  |
| 1346 | UTI |  |  | X |  |  |  |  |  |  |
| 1347 | UTI |  |  | X |  |  |  |  |  |  |
| 1348 | UTI |  | X*^P^* | X |  |  |  | X |  |  |
| 1354 | UTI |  |  | X |  |  |  |  |  |  |
| 1356 | UTI |  |  | X |  |  |  |  |  |  |
| 1358 | UTI |  |  | X |  |  |  |  |  |  |
| 1359 | UTI |  |  | X |  |  |  |  |  |  |
| 1360 | UTI |  | X | X |  |  |  |  |  |  |
| 1362 | UTI |  |  | X |  |  |  |  |  |  |
| 1526 | UTI | X*^P^* | X | X |  |  |  | X*^P^* |  | X |
| 1727 | UUI |  | X*^P^* | X |  |  |  |  |  |  |
| 2019 | UUI |  |  | X |  |  |  |  |  |  |
| 2055 | UUI |  |  | X |  |  |  |  |  |  |
| 2328 | UUI |  |  | X |  |  |  |  |  |  |
| 3538 | UUI | X | X*^P^* | X |  | X |  |  |  |  |
| 3641 | UUI | X | X*^P^* | X |  |  |  | X |  | X |
| 3643 | UUI |  | X*^P^* | X |  |  |  |  |  |  |
| 4656 | UTI |  | X*^P^* | X |  |  |  |  |  |  |
| 4716 | UUI |  |  | X |  |  |  |  |  |  |
| 4746 | UUI |  |  | X |  |  |  |  |  |  |
| 5337 | UUI |  |  | X |  |  |  |  |  |  |
| 5814 | UUI | X*^P^* | X | X |  |  |  | X*^P^* |  | X |
| 5924 | UTI | X | X*^P^* | X |  |  |  | X | X | X |
| 5978 | UTI | X*^P^* | X | X |  |  |  | X*^P^* | X | X |
| 6454 | no LUTS |  |  | X |  |  |  |  |  |  |
| 6471 | UTI |  |  | X |  |  |  |  |  |  |
| 6611 | no LUTS |  |  | X |  |  |  |  |  |  |
| 6653 | UTI | X |  | X |  |  |  | X | X | X |
| 6655 | UUI |  |  | X |  |  |  |  |  |  |
| 6713 | no LUTS | X | X | X |  |  |  | X |  |  |
| 6721 | UTI | X |  | X |  |  |  | X | X | X |
| 6890 | UUI |  |  | X |  |  |  |  |  |  |
| 7431 | UTI |  |  | X |  |  |  |  |  |  |

*P*: antibiotic resistance gene carried by a plasmid. Symptom abbreviations: UTI = urinary tract infection; OAB = overactive bladder symptoms; UUI = urgency urinary incontinence; and no LUTS = no lower urinary tract symptoms.
