# Supplementary material for: Genomic Survey of E. coli From the Bladders of Women With and Without Lower Urinary Tract Symptoms
Source: Front Microbiol. 2020 Sep 4;11:2094. doi: 10.3389/fmicb.2020.02094 (PMC7500147; doi:10.3389/fmicb.2020.02094)
Supplement: Supplementary file 6 [file Table_6.DOCX]

**Supplemental Table 6. Antibiotic resistance to fosfomycin (FOF), ciprofloxacin (CIP), amoxicillin with clavulanic acid (AMC), sulfamethoxazole-trimethoprim (SXT), and cefpodoxime (CPD) by strain. Resistance (R), Intermediate (I), and Sensitive (S) are determined according to the manufacturer’s published ranges. Red font indicates that the isolate’s genome analysis (Supplemental Table 5) identified genes associated with resistance for the particular antibiotic class.**

| **Strain** | **Participant Symptom** | **FOF** | **CIP** | **AMC** | **SXT** | **CPD** |
| --- | --- | --- | --- | --- | --- | --- |
| 103 | OAB | S | R | S | R | R |
| 149 | OAB | S | S | S | R | S |
| 276 | OAB | S | I | I | I | R |
| 527 | OAB | S | R | I | R | R |
| 731 | OAB | S | S | S | S | S |
| 906 | UTI | S | R | I | R | R |
| 923 | UTI | I | S | S | I | I |
| 928 | no LUTS | S | R | I | R | S |
| 931 | UTI | S | R | R | R | S |
| 933 | no LUTS | S | R | I | R | S |
| 934 | UTI | I | R | S | R | S |
| 939 | no LUTS | S | R | I | R | R |
| 949 | UTI | I | I | S | R | I |
| 1012 | UTI | S | S | I | R | S |
| 1091 | UTI | S | I | I | R | S |
| 1093 | UTI | S | S | I | I*^a^* | S |
| 1160 | UTI | S | I | S | R | I |
| 1161 | UTI | S | R | I | R | R |
| 1162 | UTI | S | I | S | R*^a^* | R |
| 1180 | UTI | S | S | S | S | R |
| 1193 | UTI | S | S | S | S*^a^* | S |
| 1195 | UTI | S | S | S | R | I |
| 1202 | UTI | S | I | I | I | I |
| 1220 | UTI | S | S | S | S | I |
| 1221 | UTI | S | S | S | S*^a^* | R |
| 1223 | UTI | S | S | S | R | R |
| 1225 | UTI | S | S | S | R | S |
| 1228 | UTI | S | R | S | R | S |
| 1229 | UTI | S | S | I | S*^a^* | S |
| 1284 | UTI | S | I | I | R | R |
| 1285 | UTI | S | S | I | I | R |
| 1335 | UTI | S | S | S | S | S |
| 1337 | UTI | S | I | S | S | I |
| 1346 | UTI | S | S | I | I | I |
| 1347 | UTI | S | I | S | S | I |
| 1348 | UTI | S | S | S | S*^a^* | S |
| 1354 | UTI | S | R | I | S | S |
| 1356 | UTI | S | S | S | S | R |
| 1358 | UTI | S | S | S | S | R |
| 1359 | UTI | S | R | S | I | S |
| 1360 | UTI | S | I | I | I | R |
| 1362 | UTI | S | R | S | R | I |
| 1526 | UTI | S | S | I | R | S |
| 1727 | UUI | S | S | S | S | I |
| 2019 | UUI | S | S | R | R | S |
| 2055 | UUI | S | R | R | R | S |
| 2328 | UUI | S | S | R | R | I |
| 3538 | UUI | S | I | S | S | R |
| 3641 | UUI | S | S | R | R | S |
| 3643 | UUI | S | I | R | R | R |
| 4656 | UTI | S | I | I | I | S |
| 4716 | UUI | S | S | S | I | S |
| 4746 | UUI | S | R | S | R | S |
| 5337 | UUI | S | S | S | I | S |
| 5814 | UUI | S | S | S | R | S |
| 5924 | UTI | S | I | I | S | S |
| 5978 | UTI | S | S | R | R | I |
| 6454 | no LUTS | S | R | I | I | S |
| 6471 | UTI | S | I | I | I | I |
| 6611 | no LUTS | S | S | I | S | S |
| 6653 | UTI | S | S | I | R | S |
| 6655 | UUI | S | S | S | I | I |
| 6713 | no LUTS | S | S | S | I*^a^* | S |
| 6721 | UTI | S | S | S | I | S |
| 6890 | UUI | S | I | R | R | S |
| 7431 | UTI | S | I | S | R | S |

*a*: A gene associated with sulfamethoxazole resistance was identified but a gene associated with trimethoprim resistance was not. Symptom abbreviations: UTI = urinary tract infection; OAB = overactive bladder symptoms; UUI = urgency urinary incontinence; and no LUTS = no lower urinary tract symptoms.
