# Supplementary material for: Genomic Survey of E. coli From the Bladders of Women With and Without Lower Urinary Tract Symptoms
Source: Front Microbiol. 2020 Sep 4;11:2094. doi: 10.3389/fmicb.2020.02094 (PMC7500147; doi:10.3389/fmicb.2020.02094)
Supplement: Supplementary file 7 [file Table_7.DOCX]

**Supplemental Table 7. Most similar genomes in NCBI’s nr/nt database to the 66 bladder *E. coli* isolates.**

| **Strain** | **Participant Symptom** | **BLAST Hit Strain** | **BLAST hit Accession #** | **Query Cov** | **%ID** | **Source** |
| --- | --- | --- | --- | --- | --- | --- |
| 103 | OAB | MS14385 | LR130555 | 91 | 100 | human; blood; sepsis |
| 149 | OAB | ECONIH5 | CP026202 | 85 | 99.99 | hospital wastewater |
| 276 | OAB | O18:H1 strain CFSAN067215 | CP028320 | 93 | 99.98 | human; unknown |
| 527 | OAB | CREC-532 | CP024830 | 83 | 99.98 | human; urine |
| 731 | OAB | 317 | CP023357 | 97 | 99.99 | canine; urine; UTI |
| 906 | UTI | JJ1886 | CP006784 | 96 | 99.98 | human; urine; sepsis |
| 923 | UTI | 600468 | CP025849 | 91 | 99.4 | human; stool; diarrhea |
| 928 | no LUTS | MCJCHV-1 | CP030111 | 94 | 100 | human; CSF; neonatal meningitis |
| 931 | UTI | UMN026 | CU928163 | 88 | 99.99 | human; urine; UTI |
| 933 | no LUTS | D8 | CP010151 | 86 | 99.98 | canine; stool |
| 934 | UTI | UMN026 | CU928163 | 91 | 100 | human; urine; UTI |
| 939 | no LUTS | Nissle 1917 | CP022686 | 93 | 99.99 | human; stool |
| 949 | UTI | UMN026 | CU928163 | 93 | 100 | human; urine; UTI |
| 1012 | UTI | Ecol_743 | CP015069 | 93 | 99.99 | human; either intra-abdominal infection or UTI |
| 1091 | UTI | Eco889 | CP015159 | 90 | 100 | human; urine |
| 1093 | UTI | UMN026 | CU928163 | 92 | 99.99 | human; urine; UTI |
| 1160 | UTI | MCJCHV-1 | CP030111 | 88 | 99.99 | human; CSF; neonatal meningitis |
| 1161 | UTI | Ecol_656 | CP018979 | 94 | 100 | human; unknown |
| 1162 | UTI | NGF1 | CP016007 | 86 | 99.95 | mouse; gut floura |
| 1180 | UTI | KSC9 | CP018323 | 96 | 99.99 | pig; stool |
| 1193 | UTI | D8 | CP010151 | 89 | 99.99 | canine; stool |
| 1195 | UTI | BR43-DEC | CP035377 | 84 | 99.46 | human; urine |
| 1202 | UTI | PAR | CP012379 | 86 | 99.22 | cockatoo; stool |
| 1220 | UTI | ECONIH2 | CP014667 | 98 | 99.97 | human; stool; immunodeficiency |
| 1221 | UTI | Mt1B1 | CP028714 | 86 | 99.99 | mouse; stool |
| 1223 | UTI | MS14387 | LR130564 | 92 | 99.99 | human; blood; sepsis |
| 1225 | UTI | ST2747 | CP007392 | 91 | 98.63 | human; stool; UTI |
| 1228 | UTI | AR_0055 | CP021935 | 98 | 100 | unknown |
| 1229 | UTI | ATCC 700415 | CP022609 | 95 | 99.94 | human; urine; UTI |
| 1284 | UTI | AR_0058 | CP021689 | 93 | 100 | unknown |
| 1285 | UTI | FDAARGOS_144 | CP014111 | 94 | 99.99 | human; urine; UTI |
| 1335 | UTI | 746 | CP023353 | 88 | 99.92 | canine; urine; UTI |
| 1337 | UTI | 746 | CP023353 | 88 | 99.92 | canine; urine; UTI |
| 1346 | UTI | 1190 | CP023386 | 95 | 99.99 | canine; urine; UTI |
| 1347 | UTI | 1190 | CP023386 | 95 | 99.94 | canine; urine; UTI |
| 1348 | UTI | MCJCHV-1 | CP030111 | 89 | 99.98 | human; CSF; neonatal meningitis |
| 1354 | UTI | 1190 | CP023386 | 96 | 99.94 | canine; urine; UTI |
| 1356 | UTI | 1190 | CP023386 | 96 | 99.99 | canine; urine; UTI |
| 1358 | UTI | ST540 | CP007265 | 84 | 99.26 | human; stool; UTI |
| 1359 | UTI | 1190 | CP023386 | 96 | 99.99 | canine; urine; UTI |
| 1360 | UTI | D i2 | CP002211 | 93 | 99.98 | human; stool |
| 1362 | UTI | RM9387 | CP009104 | 87 | 99.42 | cattle; stool |
| 1526 | UTI | ECONIH2 | CP014667 | 98 | 99.99 | human; stool; immunodeficiency |
| 1727 | UUI | 2017C-4173W12 | CP030768 | 89 | 99.99 | unknown |
| 2019 | UUI | S1 | CP010226 | 83 | 99.98 | forest soil |
| 2055 | UUI | May-29 | CP027373 | 90 | 99.51 | human; stool |
| 2328 | UUI | 1303 | CP009166 | 93 | 99.98 | cattle; udder acute mastitis |
| 3538 | UUI | Ecol_656 | CP018979 | 96 | 100 | human; unknown |
| 3641 | UUI | UMN026 | CU928163 | 96 | 100 | human; urine; UTI |
| 3643 | UUI | Combat2C1 | CP019243 | 93 | 100 | human; urine; UTI |
| 4656 | UTI | NCTC11151 | LR134031 | 89 | 99.99 | unknown |
| 4716 | UUI | O18:H1 strain CFSAN067215 | CP028320 | 89 | 99.97 | human; unknown |
| 4746 | UUI | M16807 | CP031256 | 94 | 99.94 | human; unknown |
| 5337 | UUI | 1190 | CP023386 | 95 | 100 | canine; urine; UTI |
| 5814 | UUI | K-15KW01 | CP016358 | 97 | 99.99 | human; stool |
| 5924 | UTI | MCJCHV-1 | CP030111 | 95 | 100 | human; CSF; neonatal meningitis |
| 5978 | UTI | MCJCHV-1 | CP030111 | 96 | 100 | human; CSF; neonatal meningitis |
| 6454 | no LUTS | SF-166 | CP012633 | 95 | 99.97 | human; blood; sepsis |
| 6471 | UTI | ST2747 | CP007392 | 87 | 98.6 | human; stool; UTI |
| 6611 | no LUTS | UTI89 | CP000243 | 95 | 100 | human; urine; UTI |
| 6653 | UTI | 2017C-4173W12 | CP030768 | 93 | 99.99 | unknown |
| 6655 | UUI | 317 | CP023357 | 92 | 99.97 | canine; urine; UTI |
| 6713 | no LUTS | PA458 | CP021288 | 95 | 99.99 | human; blood; sepsis |
| 6721 | UTI | 2017C-4173W12 | CP030768 | 93 | 99.99 | unknown |
| 6890 | UUI | 317 | CP023357 | 92 | 99.97 | canine; urine; UTI |
| 7431 | UTI | 2017C-4173W12 | CP030768 | 93 | 99.99 | unknown |

Symptom abbreviations: UTI = urinary tract infection; OAB = overactive bladder symptoms; UUI = urgency urinary incontinence; and no LUTS = no lower urinary tract symptoms.
