# Supplementary material for: Genomic Survey of E. coli From the Bladders of Women With and Without Lower Urinary Tract Symptoms
Source: Front Microbiol. 2020 Sep 4;11:2094. doi: 10.3389/fmicb.2020.02094 (PMC7500147; doi:10.3389/fmicb.2020.02094)
Supplement: Supplementary file 8 [file Table_8.DOCX]

**Supplemental Table 8. Other species identified in the urobiomes containing the *E. coli* isolates.**

| **Strain** | **Participant Symptom** | **Other Identified Species (CFU/mL)** |
| --- | --- | --- |
| 103 | OAB |  |
| 149 | OAB | *C. imitans* (70); *C. coyleae* (20); *P. aeruginosa* (10); *A. cumminsii* (50); *A. neuii* (10); *O. urethralis* (10); *S. warneri* (10) |
| 276 | OAB | *A. urinae* (10); *A. schaalii* (200); *G. vaginalis* (80); *S. anginosus* (20); *M. luteus* (10); *C. tuberculostearicum* (30); *S. epidermidis* (20); *S. capitis* (40) |
| 527 | OAB | *S. anginosus* (10); *A. radingae* (40); *A. urinae* (30); *G. vaginalis* (1500) |
| 731 | OAB | *S. anginosus* (80); *A. oris* (200); *L. iners* (500); *C. tuscaniense* (10); *L. gasseri* (80) |
| 906 | UTI | *L. gasseri* (100); *S. epidermidis* (10) |
| 923 | UTI | *R. dentocariosa* (10); *L. gasseri* (20); *A. adontolyticus* (10) |
| 928 | no LUTS | *S. pneumoniae/mitis/oralis* (40) |
| 931 | UTI | *S. aureus* (100000) |
| 933 | no LUTS | *S. hominis* (1500); *A. turicensis* (1000); *S. epidermidis* (300); *S. salivarius* (100); *A. schaalii* (50); *C. imitans* (50); *A. europaeus* (100); *A. urogenitalis* (40); *S. aureus* (10); *S. sanguinis* (10); *A. radingae* (60) |
| 934 | UTI | *E. faecalis* (500); *S. haemolyticus* (50); *L. crispatus* (200); *S. aureus* (500); *S.* *oralis/pneumoniae* (200); *S. epidermidis* (60) |
| 939 | no LUTS |  |
| 949 | UTI |  |
| 1012 | UTI | *S. agalactiae* (100); *C. amycolatum* (20) |
| 1091 | UTI | *S. capitis* (10) |
| 1093 | UTI | *L. iners* (350) |
| 1160 | UTI |  |
| 1161 | UTI |  |
| 1162 | UTI |  |
| 1180 | UTI |  |
| 1193 | UTI | *L. gasseri* (10000); *L. jensenii* (10000) |
| 1195 | UTI | *L. jensenii* (10000); *L. crispatus* (5000); *C. albicans* (1000) |
| 1202 | UTI | *S. epidermidis* (1000); *L. jensenii* (100000); *S. lugdunensis* (1000); *A. neuii* (200); *S. anginosus* (500); *L. fermentum* (500); *S. hominis* (200); *S. haemolyticus* (1000); *L. iners* (100000); *C. glucuronolyticum* (1000); *G. vaginalis* (100000); *S. warneri* (20); *C. amycolatum* (1000); *S. caprae* (200); *A. turicensis* (10000); *P. avidum* (500) |
| 1220 | UTI | *L. gasseri* (500); *E. faecalis* (300) |
| 1221 | UTI | *G. vaginalis* (100000); *L. gasseri* (10000); *A. urinae* (1000); *S. epidermidis* (10); *S. simulans* (10); *C. albicans* (100) |
| 1223 | UTI | *S. mitis* (10000) |
| 1225 | UTI | *S. salivarius* (10000); *G. vaginalis* (100000); *S. mitis/oralis* (5000); *B. breve* (1000); *S. parasanguinis* (100); *A. turicensis* (200); *S. simulans* (5000); *C. urealyticum* (500); *C. amycolatum* (100); *A. cumminsii* (200); B*. ravenspurgense* (5000); Gram + cocci #1 (5000); Gram + cocci #2 (1000); Gram + rods (1000) |
| 1228 | UTI | *K. pneumoniae* (100000); *E. faecalis* (10) |
| 1229 | UTI | *S. anginosus* (1000); *L. iners* (10000); *G. vaginalis* (10000); *L. crispatus* (2000) |
| 1284 | UTI | *A. urinae* (10000); *T. bernardiae* (500); *L. iners* (1000); *C. riegelii* (100); *F. hominis* (100); *S. epidermidis* (10); *C. coyleae* (40) |
| 1285 | UTI | *L. jensenii* (20) |
| 1335 | UTI | *A. turicensis* (100) |
| 1337 | UTI | *E. faecalis* (1000); *L. gasseri* (2000); *C. amycolatum* (20); *A. neuii* (150); *S. hominis* (10) |
| 1346 | UTI |  |
| 1347 | UTI | *S. anginosus* (20) |
| 1348 | UTI |  |
| 1354 | UTI | *S. anginosus* (10000) |
| 1356 | UTI | *S. anginosus* (10) |
| 1358 | UTI | *S. anginosus* (100) |
| 1359 | UTI |  |
| 1360 | UTI | *S. anginosus* (10) |
| 1362 | UTI |  |
| 1526 | UTI |  |
| 1727 | UUI | *K. pneumoniae* (10000) |
| 2019 | UUI | *G*. species (20000); *G. vaginalis* (500); *L. jensenii* (5000); *L. iners* (5000) |
| 2055 | UUI | *A. urinae* (35); *E. faecalis* (2); *L. gasseri* (33); *L. rhamnosus* (1); *S. epidermidis* (3); Unkn (5) |
| 2328 | UUI |  |
| 3538 | UUI |  |
| 3641 | UUI | *L. iners* (100000) |
| 3643 | UUI | *A. omnicolens* (10); *C. coyleae* (10); *C. imitans* (10); *C. riegelii* (10); *C*. species (10); *L. amylovorus* (50); *L. gasseri* (50); *L. jensenii* (10); *S. anginosis* (10) |
| 4656 | UTI | *A. urinae* (10); *G. vaginalis* (10); *K. pneumoniae* (1000000) |
| 4716 | UUI | *A. neuii* (10); *G. vaginalis* (3000) |
| 4746 | UUI |  |
| 5337 | UUI | *A. radingae* (20); *S. anginosus* (2510); Unkn (440) |
| 5814 | UUI | *L. delbrueckii* (10000) |
| 5924 | UTI | *S. agalactiae* (10000) |
| 5978 | UTI | *G. vaginalis* (100000); *L. iners* (100000); *S. mitis* (50000) |
| 6454 | no LUTS | *L. delbrueckii* (10000) |
| 6471 | UTI |  |
| 6611 | no LUTS | *C. coyleae* (10); Unkn (10) |
| 6653 | UTI | *C. lusitaniae* (20); *L. gasseri* (30) |
| 6655 | UUI | *S. constellatus* (40) |
| 6713 | no LUTS | *L. crispatus* (20) |
| 6721 | UTI | *L. crispatus* (20) |
| 6890 | UUI | *M. luteus* (10); *S. oralis* (20) |
| 7431 | UTI | *C. lusitaniae* (40); *L. gasseri* (2000) |

“Unkn” indicates that the species could not be identified by MALDI-TOF. Symptom abbreviations: UTI = urinary tract infection; OAB = overactive bladder symptoms; UUI = urgency urinary incontinence; and no LUTS = no lower urinary tract symptoms.
